# Supplementary figures and images for: The CRISPR-Cas systems were selectively inactivated during evolution of Bacillus cereus group for adaptation to diverse environments
Source: ISME J. 2020 Mar 4;14(6):1479–93. doi: 10.1038/s41396-020-0623-5 (PMC7242445; doi:10.1038/s41396-020-0623-5)

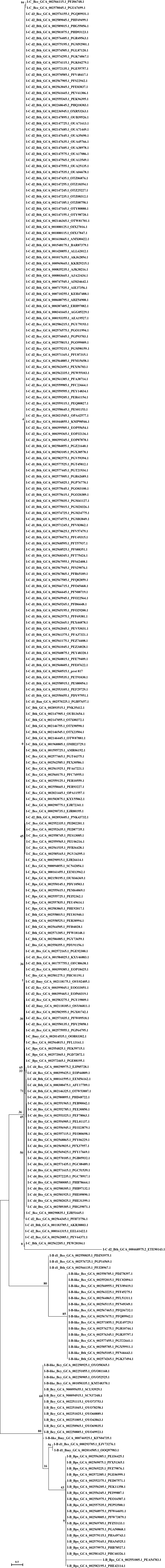

Supplement: Supplementary file 4 — Fig. S3 [file 41396_2020_623_MOESM4_ESM.tif]

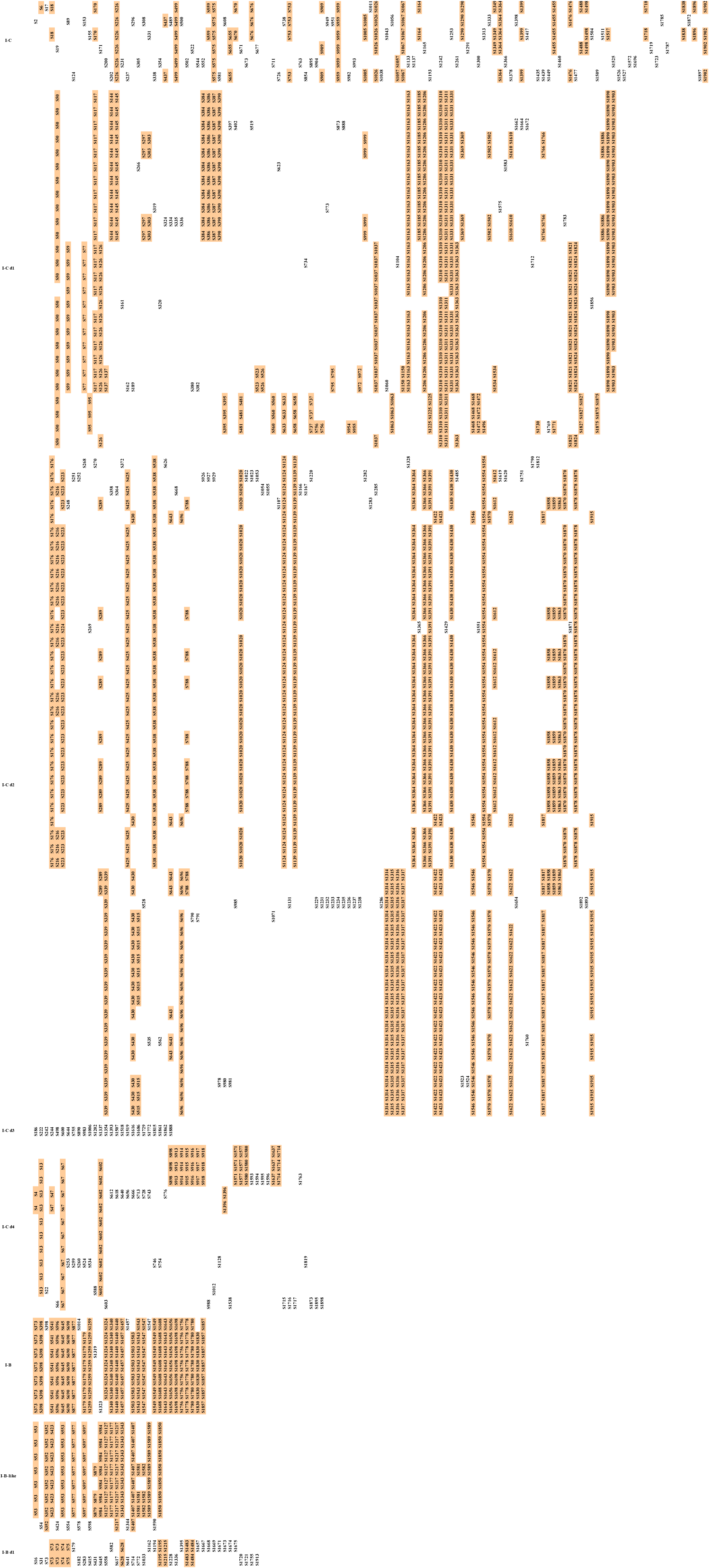

Supplement: Supplementary file 5 — Fig. S4 [file 41396_2020_623_MOESM5_ESM.tif]
