# Supplementary material for: The CRISPR-Cas systems were selectively inactivated during evolution of Bacillus cereus group for adaptation to diverse environments
Source: ISME J. 2020 Mar 4;14(6):1479–93. doi: 10.1038/s41396-020-0623-5 (PMC7242445; doi:10.1038/s41396-020-0623-5)
Supplement: Supplementary file 15 — Manuscript_revised version [file 41396_2020_623_MOESM15_ESM.docx]

**The CRISPR-Cas systems were selectively inactivated during evolution of *Bacillus cereus* group for adaptation to diverse environments**

**Running title:** Inactivation of CRISPR-Cas systems in Bc group

Ziqiang Zheng^1^, Yulan Zhang^1^, Zhiyu Liu^1^, Zhaoxia Dong^1^, Chuanshuai Xie^1^, Alejandra Bravo^2^, Mario Soberón^2^, Jacques Mahillon^3^, Ming Sun^1^*, and Donghai Peng^1^*

^1^State Key Laboratory of Agricultural Microbiology, College of Life Science and Technology, Huazhong Agricultural University, Wuhan 430070, Hubei, China

^2^Instituto de Biotecnología, Universidad Nacional Autónoma de México, Apdo. postal 510-3, Cuernavaca, 62250 Morelos, Mexico.

^3^Laboratory of Food and Environmental Microbiology, Earth and Life Institute, UCLouvain, Croix du Sud, 2 - L7.05.12, B-1348 Louvain-la-Neuve, Belgium

*Corresponding authors: E-mail: D.H.P. [donghaipeng@mail.hzau.edu.cn](mailto:donghaipeng@mail.hzau.edu.cn), or M.S. m98sun@mail.hzau.edu.cn; TEL: 86-27-87283455; FAX: 86-27-87280670.**Abstract**

CRISPR-Cas systems are considered as barriers to horizontal gene transfer (HGT). However, the influence of such systems on HGT within species is unclear. Also, little is known about the impact of CRISPR-Cas systems on bacterial evolution at the population level. Here, using *Bacillus cereus* sensu lato as model, we investigate the interplay between CRISPR-Cas systems and HGT at the population scale. We found that only a small fraction of the strains have CRISPR-Cas systems (13.9% of 1,871), and most of such systems are defective based on their gene content analysis. Comparative genomic analysis revealed that the CRISPR-Cas systems are barriers to HGT within this group, since strains harboring active systems contain less mobile genetic elements (MGEs), have lower fraction of unique genes and also display limited environmental distributions than strains without active CRISPR-Cas systems. The introduction of a functional CRISPR-Cas system into a strain lacking the system resulted in reduced adaptability to various stresses and decreased pathogenicity of the transformant strain, indicating that *B. cereus* group strains could benefit from inactivating such systems. Our work provides a large-scale case to support that the CRISPR-Cas systems are barriers to HGT within species, and that in the *B. cereus* group the inactivation of CRISPR-Cas systems correlated with acquisition of MGEs that could result in better adaptation to diverse environments.

**Introduction**

Clustered regularly interspaced short palindromic repeats (CRISPR) and its associated proteins (Cas) systems function as an antiviral defense pathway in prokaryotes [[1](#_ENREF_1), [2](#_ENREF_2)]. A typical CRISPR-Cas system consists of three functional components: an operon containing a set of *cas* genes, a leader sequence, and a CRISPR DNA array. The *cas* genes are usually required for new spacer acquisition, target recognition and degradation of foreign nucleic acids [[3](#_ENREF_3), [4](#_ENREF_4)]. The classification of CRISPR-Cas systems is based on their signature of cas genes, the organization of *cas* operons, and the direct repeats present in the CRISPR arrays [[5](#_ENREF_5)]. Six types of CRISPR-Cas systems have been characterized: types I, III and IV of class 1 [[6](#_ENREF_6)], and types II, V and VI of class 2 [[7](#_ENREF_7)].

The discovery and characterization of CRISPR-Cas, especially in members of Class 2 systems, has led to a revolution in genome editing and engineering technologies [[7-9](#_ENREF_7)]. Beyond its immune function, a number of recent observations revealed unexpected roles for CRISPR-Cas systems in gene expression, autoimmunity, chromosomal segregation or rearrangement, and DNA repairing, although most of the mechanisms involved in these functions still remain unclear [[10](#_ENREF_10)]. Of particular interest was the discovery of the involvement of CRISPR-Cas systems on the regulation of bacterial virulence [[11-13](#_ENREF_11)].

The roles for CRISPR-Cas systems on horizontal gene transfer (HGT) have been widely investigated. It was primary proposed that CRISPR-Cas system may prevent the acquisition of advantageous mobile genetic elements (MGEs) [[4](#_ENREF_4)], because of its immune function against acquisition of foreign DNA. A series of studies support this hypothesis and demonstrate that CRISPR-Cas limits HGT through conjugation and transformation. For example, the CRISPR loci in *Staphylococci* counteracts multiple routes of HGT to limit the spread of antibiotic resistance genes [[4](#_ENREF_4)]. Similarly, in *S. pneumoniae* the CRISPR-Cas prevents the acquisition of virulence related genes [[11](#_ENREF_11)], and in *Enterococcus faecalis* it was reported that the potential for DNA acquisition could be maximized by attenuating its CRISPR-Cas system [[14](#_ENREF_14), [15](#_ENREF_15)]. Previously, a bioinformatic-study showed that there is no detectable inﬂuence of CRISPR-Cas systems on the HGT rate over evolutionary timescales among species [[16](#_ENREF_16)]. However, new evidence provides an opposite view, suggesting that CRISPR-Cas facilitates the HGT by transduction [[17-19](#_ENREF_17)]. For instance, the CRISPR-Cas system in *Pectobacterium atrosepticum* facilitates acquisition of non-phage genetic material by phage-mediated transduction [[18](#_ENREF_18)]. Also, it was shown that phages can recombine with spacers from CRISPR-Cas loci leading to transfer CRISPR-adjacent genes promoting HGT in *Staphylococci* [[19](#_ENREF_19)].

Despite the relevance of CRISPR-Cas systems as antiviral defense strategy in bacteria, the inﬂuence of CRISPR-Cas on HGT has remained a matter of debate. The existing studies mostly were conducted with individual strains, but these analyzes cannot truly reflect the relationship between CRISPR-Cas systems and HGT within species at the population scale. Additionally, the CRISPR-Cas systems have been proposed as important factors contributing for the evolution of virulence in several pathogens [[11](#_ENREF_11), [14-16](#_ENREF_14), [18](#_ENREF_18)], but the impact of such systems on bacterial evolution at population level is also poorly understood. The increasing availability of sequenced genomes will facilitate the study and understanding of the role interplayed among CRISPR-Cas systems and HGT, and its impact on bacterial evolution within species.

Members of the *B. cereus* group are ubiquitous and highly versatile bacteria. It has been proposed that they have evolved in ecotypes since they differ mainly in their plasmid DNA content [[20](#_ENREF_20)]. The plasmids present in this group of bacteria harbor different toxin genes, conferring diverse host specificities such as *B. thuringiensis*, that is an important entomopathogen used worldwide as an effective bio-insecticide to control various pests [[21](#_ENREF_21)]; *B. anthracis*, which cause anthrax and *B. cereus* that may cause food-borne gastroenteritis [[22](#_ENREF_22), [23](#_ENREF_23)]. The large number of available genomes in this group provides a good model to study the relationship among CRISPR-Cas systems and HGT at the population level.

In this study, we determined the prevalence, diversity and phylogenetic distribution of CRISPR-Cas systems present within 1,871 genomes from *B. cereus* group. Our results show that most of the strains analyzed did not contain any CRISPR-Cas system or harbor defective systems. Combined with large-scale comparative genomic analysis, we concluded that the CRISPR-Cas systems are barriers to HGT within this group of bacteria. Even more, we evaluated the impact of CRISPR-Cas systems at the population level, showing that in *B. cereus* group the inactivation of such systems correlates with MGEs acquisition that could provide genetic traits for better adaptation to diverse environments.

**Materials and methods**

**Identification of CRISPR-Cas systems among *B. cereus* group**

The genome sequences of the 1,871 strains from *B. cereus* group including their chromosome and plasmids sequences were retrieved from GenBank (ftp://ftp.ncbi.nlm.nih.gov/genomes). Cas proteins prediction was performed through BLASTP using genome-wide amino acid sequences of the 1,871 strains against known Cas proteins, with an identity threshold of 30% and dual coverage over 60% [[24](#_ENREF_24)]. The predicted Cas proteins and spacers were verified with CRISPR-finder [[25](#_ENREF_25)]. Classification of CRISPR-Cas systems was based on the *cas* gene organization as previously reported [[6](#_ENREF_6), [26](#_ENREF_26)]. The information of the 260 *B. cereus* group strains found to contain CRISPR-Cas systems are listed in Table S1.

**Phylogenetic analysis**

The sequence of Cas7 proteins were clustered by using the MCL algorithm with an inflation value of 1.5, after an all-against-all BLASTP search with an E value of <10^5^ [[27](#_ENREF_27)]. The single-copy core protein sequences of each cluster were aligned with MUSCLE [[28](#_ENREF_28)]. All alignments were concatenated using an in-house Perl script. ML phylogenetic tree was constructed using FastTree software [[29](#_ENREF_29)] with bootstrap support values calculated from 1,000 replicates.

**Analysis of environmental niches distribution**

Information of the original ecological niches of 423 selected strains from the original 1,871 strains was collected from NCBI (<https://www.ncbi.nlm.nih.gov/>) or from published papers (Table S2). We included a total of 423 strains which information on niche isolation was available. The niches include targets, hosts and/or sources, which were classified into six major groups, including: the animal-associated group, that is composed by fish, insects, mouse, nematode and earthworm subgroups; the human-associated group, that is composed by human and human-excrement subgroups; the plant-associated group, that is composed by plant, plant core, plant leaf, plant rhizosphere and plant root subgroups; the fungi-associated group; the soil-associated group; and others group, which includes food, industrial product and waste water subgroups.

**Prediction of transposases, plasmid essential replication proteins and prophage proteins**

The protein sequences of the 423 selected stains described above were constructed as target sequence database. A complete profile of transposases downloaded from TnpPred [[30](#_ENREF_30)] was searched against this database by using HMMER [[31](#_ENREF_31)] with sequence and domain threshold E values of <10^5^. The predicted transposases are listed in Table S3. Replication essential proteins [[32](#_ENREF_32)] were used to predict plasmids (TXT S1). TubZ model (TXT S2) is obtained from the Pfam database [[33](#_ENREF_33)], and the TubZ protein sequences were obtained with the same HMMER search command as transposase prediction. The other sequences of essential plasmid replication proteins were obtained using BLASTP with an identity threshold above 30% and a dual coverage over 60%. All the essential plasmid replication proteins predicted are listed in Table S4. Prophage elements were predicted by PHASTER [[34](#_ENREF_34)] and are listed in Table S5.

**Unique gene diversity calculations**

The presence and absence of genes in the 423 selected genomes described above was determined by using the Roary software [[27](#_ENREF_27)]. The genes shared by any two strains refer to the core genes, the sum of genes contained by both strains refers the total genes, and the difference of core genes and total genes refers to the unique genes. The ratio of number of unique genes against total genes of each genome pair refers the unique gene diversity value. The unique gene diversity values were calculated in groups of strains with complete, incomplete and none CRISPR-Cas systems that are listed in Table S6.

**Growth conditions for bacterial strains and nematode *Caenorhabditis elegans***

Bacterial strains and plasmids used in this work are listed in Table S7. All *B. cereus* group strains were grown at 28^◦^C in Luria-Bertani (LB) medium and the competent cells were transformed by electroporation [[36](#_ENREF_36)]. *Escherichia coli* strains were grown at 37^◦^C in LB medium. The nematode *C. elegans* Bristol N2 strain was grown at 20^◦^C as previously described [[37](#_ENREF_37)].

**Biofilm formation assays**

Biofilm formation was evaluated in 96-well plates as described before [[38](#_ENREF_38)].

**Determination of sporulation rate**

Overnight bacterial cultures (100 µl, OD_600_=0.3) were spread on solid LB-agar plates and allowed them to sporulate for 5 days at 28°C. The spores were harvested and suspended in 1.5 ml ice-cold 0.85% NaCl, and two aliquots of 500 µl of spore suspensions were prepared. The aliquot “*a*” was heated at 80°C for 10 min to kill the vegetative cells and then kept on ice for 10 min; while the aliquot “*b*” was just kept on ice. Serial 10-fold dilutions were prepared and 100 µl of each dilution was plated onto LB-agar plates. Growing colonies were counted after 24 h of incubation, and the sporulation rates were calculated as the percentage of colony forming units (CFU) of aliquot “*a*” (CFU-*a*) divide by the CFU of aliquot “*b*” (CFU-*b*) for each strain.

**Salt tolerance assay**

Overnight bacterial cultures in LB medium (100 µl, OD_600_=0.3) were spread on solid LB-agar medium supplemented with 1, 2, 4 or 6% NaCl. The bacterial colonies were counted after 12 h, and the bacterial surviving rates on these media were calculated using the 1% NaCl medium as reference.

**pH tolerance assay**

One ml sample of the bacterial cultures in LB medium (OD_600_=0.6) were collected. The pellet was washed with sterilized ddH_2_O three times and suspended in 1 ml sterilized ddH_2_O adjusted at pH 4.0 to 10.0, followed by incubation for 30 min with agitation at 220 rpm. The buffers used to establish the optimum pH and to maintain pH stability were as follows: sodium acetate buffer (0.2 mol/L NaAc and 0.2 mol/L HAc, pH 4.0–5.0), phosphate buffer (0.06 mol/L Na_2_HPO_4_ and 0.06 mol/L KH_2_PO_4_, pH 6.0–8.0) and Gly-NaOH buffer (0.2 mol/L glycine and 0.2 mol/L NaOH, pH 9.0–10.0) [[39](#_ENREF_39)]. The pellets were washed with sterilized ddH_2_O three times, suspended in 0.1 ml sterilized ddH_2_O again and spread on the solid LB-agar medium. The bacterial colonies were counted after 12 h incubation. The surviving rates of bacteria were calculated, using suspension incubated at pH 7.0 as reference.

***In vivo* colonization assays**

The *in vivo* colonization assays for *B. cereus* group strains was conducted as described before [[40](#_ENREF_40)]. A dose of 10^3^ spores from each strain were used to feed *C. elegans* L4 worms in 96-well plates.

**Life-span assays of nematode *C. elegans***

A total of 50-100 L4 stage N2 worms were transferred to fresh NG plates spread with 30μl (OD_600_=0.3) spore/crystals mixtures of *B. thuringiensis* strains, with 25 μΜ FUDR to prevent eggs from hatching. NG plates spread with *E. coli* strain OP50 were used as controls. The worms were scored for alive/dead every 12 h at 20°C until 96 h. The survival fractions were calculated as the number of living nematodes to that of all nematodes added in each plate, and survival curves were compared using *t*-test with statistical significance set at *p* < 0.05.

**Results**

**CRISPR locus analysis and its distribution among *B. cereus* group strains**

To determine the distribution and diversity of CRISPR-Cas systems in *B. cereus* group, we retrieved from GenBank all the 1,871 whole-genome sequences available from this group. The search of CRISPR-Cas elements in the available genomes revealed that only 260 strains (*ca.* 13.9 %) contained type I CRISPR-Cas system related elements, while only one strain of *B. cytotoxicus,* AFSSA_08CEB44bac, contained type II as revealed by the presence of a Cas9-like protein (Table S1). Interestingly, no other type systems were found among these strains and most of the strains possessed only one CRISPR-Cas structure, with the exception of *B. cytotoxicus* CH_23 (Table S1). The occurrence of CRISPR-Cas system varied greatly among the ecotypes of this group (Table 1). The highest proportion was found in *B. cytotoxicus* where 86 % of the analyzed genomes (14 genomes available) contain CRISPER-Cas elements, while only one *B. anthracis* strain from a total of 188 *B. anthracis* genomes analyzed was found to contain CRISPR-Cas elements. In the case of the *B. cereus* strains, 9 % of 862 genomes analyzed showed CRISPER-Cas elements, while regarding to the *B. thuringiensis* strains, we found that 26 % of strains have CRISPR-Cas elements from a total of 445 genomes analyzed (Table 1). Interestingly, only 30 of the 252 CRISPR-Cas system loci (11 %) (8 of them could not be classified) were located on the chromosome, while the majority (88%) and were plasmid-borne (Table S1), suggesting that these CRISPR-Cas systems were mainly acquired by HGT.

Based on the signature genes and gene arrangements, two different intact subtypes I were identified (subtypes I-B and I-C), and one variant of subtype I-B system was also found (named subtype I-B-like) (Table S1 and Fig. 1A). In subtype I-B like, the *cas8* is replaced by *casX* encoding a hypothetical protein (Fig 1A). Among these complete systems, subtype I-C was the most abundant, which accounted for 18.85% of all identified systems (Fig 1B), and it was found in 49 strains across five ecotypes of bacteria (Table S1, Fig. 1B). The subtype I-B system was only present in 27 strains, while the I-B-like system was found in 20 strains. The incomplete systems accounted for 63.08% of all identified systems (Fig. 1B and detailed information in Table S1).

The number of spacers varied from 10 to 44 for subtype I-C, 0 to 32 for subtype I-B, and 10 to 34 for I-B like system (Table S1). A total of 4,788 spacers were identified covering 1,916 unique types (Dataset 1). However, only 701 out of the 1,916 unique types of spacer have targets in the GenBank nucleotide sequence database (Dataset 2). Among them, 436 matched with sequences of plasmids, 57 with bacteriophage genes, and 288 with chromosome sequences (Dataset 2 and Fig. S1). Most of the spacers have sequence similarity with plasmids and phages (Fig. S2), consistent with the CRISPR-Cas function as a protective immune system against acquiring foreign nucleic acids. The high number of spacer that matched with chromosome sequences is very interesting. Some of the targets in the chromosome seemed to be conserved, and the three most common targets are collagen-like protein (30 hits), MoxR family ATPase (18 hits), and cell division protein FtsK (15 hits) (Dataset 2 and Table S8). These 288 unique spacers that matched chromosome sequences, include 34 self-targeting spacers (Fig. S2 and Dataset 1), which may raise an issue of auto-immunity in the host. The incorporation of self-chromosomal DNA into the CRISPR-Cas systems has been documented in bacteria and it was proposed that this may induce autoimmune responses resulting in high fitness cost [[41](#_ENREF_41)]. Thus, it was proposed that the autoimmune responses could explain the high frequency and abundance of incomplete CRISPR-Cas systems across some prokaryotes [[41](#_ENREF_41)]. Moreover, there are 239 intra-species targeting and 85 inter-species targeting spacers (Fig. S2 and Dataset 1). It has been reported that the inter-species targeting spacers have a negative effect on mating success in *Archaea* and may limit inter-species HGT [[42](#_ENREF_42)].

**Most CRISPR-Cas systems display a genetic inactivation phenomenon**

Sixty three % (164 of 260) of the identified CRISPR-Cas systems were considered as not functional based on the different gene deletions observed in the CRISPR-Cas systems of *B. cereus* group (Table S1). These defective systems derived from the intact subtypes I-C or I-B, displaying deletion of certain *cas* genes (Fig. 2A). The exception was the defective type II system that was identified in *B. cytotoxicus* strain AFSSA_08CEB44bac (Table S1). Based on the Cas protein composition and organization, these 164 defective systems were divided into five variants: I-C d1, I-C d2, I-C d3, I-C d4 derived from intact subtype I-C, and I-B d1 derived from intact subtype I-B or I-B like (Fig. 2A and Table S1). Specifically, in variant I-C d1 (identified in 47 strains), the *cas4* and *cas1* genes of intact subtype I-C were replaced by two *csd2* genes and one regulator *albA* gene, that encode proteins which have different functions than Cas4 and Cas1. We performed BlastP analysis of these *csd2* and *albA* genes finding that *csd2* is related to a CRISPR-associated protein, with high similarity to Cas7 a backbone protein that forms nucleic acid cleavage complexes. While the *albA* gene encodes a transcriptional regulator, containing a putative DNA-binding domain (AlbA_2), which function is unknown. Variant I-C d2 (identified in 87 strains) lacks *cas1*, *cas2* and *cas4* genes. Variant I-C d3 (identified in only one strain) showed a truncated CRISPR-Cas system lacking *cas3*, *cas5*, and the N-terminal end of *cas8* compared to the intact subtype I-C. Variant I-C d4 (identified in 24 strains) lost most of the elements and only has *cas5*, *cas8* and *cas7* genes. Similarly, variant I-B d1 (identified in 5 strains) showed only *cas3*, *cas5* and *cas7* genes with deletions of CRISPR-Cas system elements at both upstream and downstream regions of the intact subtype I-B locus (Fig. 2A).

To understand the pathways that may have lead to these defective systems, a phylogenetic tree was constructed based on Cas7 protein sequence, since this protein is present in all defective variants and intact systems. As shown in Fig. 2B, the systems are grouped in two major clades, one containing all subtype I-C and I-C variants while the other contains all subtype I-B, I-B like, and I-B variants (detailed shown in Fig. S3). Moreover, inside each major clade, with few exceptions, most systems in the same subtype or same variants formed separate branches. These results strongly suggest that the defective CRISPR-Cas systems evolved from intact systems, with a lineage relationship respective to the degeneration pathways of I-C and I-B subtypes (Fig. 2A).

Together, the Cas protein organization profiles and phylogenetic relationship analysis, suggest that the degeneration of CRISPR-Cas systems in the *B. cereus* group may have followed two main inactivation pathways (Fig. 2C): in the inactivation pathway for subtype I-C variants (Fig 2C), the *cas1* and *cas4* genes, which are responsible for acquiring new spacers, were replaced by *csd2* and *albA* genes (variant I-C d1). In a subsequent event, variant I-C d2 was formed, where the *csd2* and *albA* genes together with *cas2* were lost. Finally, the *cas3* gene was deleted leading to variant I-C d4 (Fig 2C). In the case of variant I-C d3, *cas3, cas 5* and *cas 8* genes were deleted (Fig 2C). This variant may be an accidental event, since it was observed in only one strain. In the inactivation pathway for subtype I-B variants (Fig 2C), replacement of *cas8* gene by *casX* resulted in subtype I-B-like, then a directly derived CRISPR-Cas system (variant I-B d1) evolved, where *cas6, cas 8, cas4, cas1* and *cas2* were deleted without further intermediate steps (Fig 2C). Interestingly, in variant I-B d1, the *cas* genes responsible for acquiring new spacers were also lost. Overall, inactivation pathway for subtype I-C variants may represent the major degeneration route of CRISPR-Cas systems in *B. cereus* group, since this was observed in 208 of the 260 strains that contain CRISPR-Cas systems (Table S1).

Spacer acquisition is one of the most important functions of CRISPR-Cas systems and the spacer diversity in CRISPR array reflects the interaction frequency and course between bacterial host and invasive nucleic acids. In *B. cereus* group, there are more spacers and higher diversity of spacer compositions in strains with intact subtype I-C system compared to those containing the I-C defective systems (Fig. 2D, Fig. S4 and Table S1). In particular, the types of spacers diminished gradually from subtype I-C to variants I-C d1-d2 and then to variant I-C d4, in accordance with the proposed deletion pathway of *cas* genes in this subtype group (Fig. 2D).

**Strains with intact CRISPR-Cas system contain restricted niche distribution, less MGEs, and less variable genes**

The CRISPR-Cas system has been proposed as barriers to HGT preventing acquisition of MGEs [[4](#_ENREF_4)]. Therefore, one possibility is that intact CRISPR-Cas system may prevent acquisition of advantageous exogenous genes to adapt to complex environments. To confirm this hypothesis, 96 genomes that contain intact CRISPR-Cas systems, 164 genomes that contain defective variant systems, and 163 genomes without any CRISPR-Cas elements (randomly selected from the 1871 genomes reported in GenBank) were analyzed regarding their content of DNA obtained by HGT.

We first analyzed the distribution of strains containing complete or incomplete systems at the population level. A maximum-likelihood (ML) phylogenetic tree based on the core genome of the above strains was constructed by FastTree. Phylogenomic analysis divided these strains into six clades (Fig. 3), which are in accordance with the population structure previously described for the *B. cereus* group [[43](#_ENREF_43)]. In addition, *B. cytotoxicus* and *B. pseudomycoides* were included into the new clades 5 and 6, respectively. Interestingly, the intact CRISPR-Cas systems can be found in all six clades, while the incomplete systems are only distributed in clades 1, 2, 3 and 4. However, in each of these clades, the distribution of strains with incomplete systems is more widely represented than that of strains containing intact systems (Fig. 3). These data indicate that the defective CRISPR-Cas systems have more widely distribution than that of the intact systems at the population level in the *B. cereus* group. Even more, strains that contain same CRISPR-Cas variant do not form separate branches in the population structure, but are scattered in each clade, indicating that inactivation of CRISPR-Cas systems are related to HGT, rather than inherited from parental lineages.

Strains of *B. cereus* group can be isolated from diverse environments as pathogenic or non-pathogenic bacteria [[44](#_ENREF_44), [45](#_ENREF_45)], making this group a powerful system for investigating the role of CRISPR-Cas systems in niche adaptability. The environmental distribution of strains was obtained for those strains where information was available regarding their isolation source (see Materials and Methods). At least 21 kinds of different environments, such as human, animal, plant, fungi, soil and others were identified (Fig. 4A). Interestingly, strains with incomplete and without CRISPR-Cas systems occupied much more diverse environments than strains with intact CRISPR-Cas systems (Fig. 4B), indicating that the CRISPR-Cas systems may limit their environmental distribution.

HGT plays important roles in bacterial adaptation to different environments [[46](#_ENREF_46)]. Inactivation of CRISPR-Cas systems could explain HGT differences of the *B. cereus* group*.* To determine the capacity of HGT, we analyzed the content of MGEs, including putative transposases, replication proteins of plasmids, and prophage elements in the different strains. The results showed that the content of putative transposases (Fig. 5A and Table S3), plasmid replication proteins (Fig. 5B and Table S4) and prophage elements (Fig. 5C and Table S5) in strains with intact CRISPR-Cas systems were significantly lower than those of strains with incomplete or lacking CRISPR-Cas systems.

Some genes, such as virulence factors are carried by mobile elements. Furthermore, genes may be able to persist in MGEs through hitch-hiking with beneficial genes or alleles ensuring high frequencies via selection of MGEs [[47](#_ENREF_47)] and transfer between organisms [[48](#_ENREF_48)]. To evaluate the direct effects of CRISPR-Cas system on HGT, we analyzed the variation of unique genes of strains with complete, incomplete and none CRISPR-Cas systems in the *B. cereus* group. These analyzes showed that variation of unique genes is significantly lower in the strains with complete CRISPR-Cas systems (n=93) than in strains with incomplete (n=164, *p*<0.0001) or none CRISPR-Cas system groups (n=135, *p*<0.05) (Fig. 5D). Together, our results suggest that the CRISPR-Cas system reduces HGT frequency within *B. cereus* group.

**Active CRISPR-Cas system displays negative impact on the adaptation of** ***B. thuringiensis* under extreme conditions**

To further test the hypothesis that bacteria with active CRISPR-Cas system may reduce their environmental adaptation, a complete subtype I-C system form *B. cereus* strain VD115 (Fig. 6A and Fig. S5) was cloned (Supplementary Text, Fig. S6). We selected to clone subtype I-C from VD115 strain to examine its function since this is a functional system (Supplementary Text, Fig.S7) and also because inactivation pathway for subtype I-C variants represents the major inactivation pathway of CRISPR-Cas systems in the *B. cereus* group (Fig. 2). We confirmed that the PAM site of VD115 subtype I-C system is TTN (Fig. 6B). We constructed derivatives vectors of pHT-304 containing synthetic VD115 spacer 1 (Table S9) with upstream 5’-TTN or four random 5’-NNN PAMs, and then transformed these vectors into strain VD115. The transformation efficiency of plasmids containing spacer 1 accompanied by 5’-TTN were significantly lower than those accompanied by non-5’-TTN (Fig. 6B), confirming that the PAM site of VD115 subtype I-C system is TTN.

Then the effect of the active CRISPR-Cas system from *B. cereus* strain VD115 on the adaptation to different environments was evaluated when transformed into a wild type *B. thuringiensis* strain YBT-1518 lacking CRISPR-Cas resulting in BMB1685 strain. We selected to work with *B. thuringiensis* YBT-1518 strain because it lacks CRISPR-Cas systems and also because it is toxic to the nematode *C. elegans* [[40](#_ENREF_40)] allowing the analysis of CRISPR-Cas on pathogenicity. We first analyzed the functionality of CRISPR-Cas system when expressed in *B. thuringiensis* BMB1685 strain. To detect if this CRISPR locus protects from exogenous DNA, we introduced synthetic spacers 1, 8, 10, 13 and 14 with the 5’-TTC PAM sites into the vector pHT-304 (Table S10). Then, these constructed plasmids and the pHT-304 vector, were transformed into strain VD115 and recombinant BMB1685. The results showed that the CRISPR locus in both strains reduced the transformation efficiency of plasmids containing spacers flanked by 5’-TTC (Fig. 6C and 6D).

The genome editing function of the cloned subtype I-C system in BMB1685 pBAC44 was also analyzed. The nematicidal *cry6Aa* gene [[49](#_ENREF_49)], which codes for a 54-kDa protein active against nematodes, was selected as target. For this purpose, a plasmid containing a small guide RNA (sgRNA) for *cry6Aa* gene was constructed as described in supplemental text (Fig S8A) and introduced into BMB1685 strain. Sixty-nine colonies were tested by PCR, and 98.5% of the colonies displayed a smaller band (568 bp) in agarose gel electrophoresis that represents the deletion in *cry6Aa* gene (Fig. S8B). The sequence of the PCR products confirmed the 198 bp deletion on *cry6Aa* gene as designed (Fig. 6E). Finally, analysis of the expression of Cry6Aa protein showed that this 54 kDa protein band was not detected in SDS-PAGE in the strains with the 198 bp deletion (Fig. 6F). Taking together, above results showed that cloned VD115 subtype I-C CRISPR-Cas system is functional in the *B. thuringiensis* transformant BMB1685 strain..

In order to analyze the effect of CRISPR-Cas system expression in the environmental adaptation of *B. thuringiensis* BMB1685 strain, we first analyzed bacterial growth in LB medium. As shown in Fig. 7A, the growth curves no differences on growth during their logarithmic growth phase were observed among YBT-1518, control BMB1684 and BMB1685 containing the complete subtype I-C system. However, after 20 h of growth, BMB1685 showed significant lower biomass till the end of the experiment. These results suggest that subtype I-C system decreases the growth of bacteria during stationary phase. In addition, the sporulation rate of BMB1685 showed a significant decrease compared to that of the *B. thuringiensis* YBT-1518 and control BMB1684 strains (Fig. 7B).

Biofilm is a community of surface-associated bacteria, which is generally composed of exopolysaccharides, proteins and some nucleic acids to protect bacteria from severe environmental conditions [[50](#_ENREF_50)]. To detect the effect of CRISPR-Cas system on biofilm formation, the biofilm formation ability of the *B. thuringiensis* BMB1685, YBT-1518 and BMB1684 strains was compared. On the basis of the OD_595_ of the soluble dye, a significant lower staining was found in the BMB1685 strain transformed with the complete subtype I-C system, indicating that CRISPR-Cas system negatively affects the biofilm formation ability (Fig. 7C and Fig. S9).

To determine the effect of CRISPR-Cas system on the tolerance to salt or pH, we tested the survival rate of cells grown on LB plates supplemented with different salt concentrations or at various pH, from pH 4 to pH 10. The results showed that bacteria without of CRISPR-Cas system grew better at 2% salt condition compared to the BMB1685 strain transformed with CRISPER-Cas system (Fig. 7D). Similarly, the growth of BMB1685 was not affected after incubation at pH 7.0 and pH 8.0 while cell survival of this strain decreased significantly after incubation at extreme acidic (pH 5.0 and pH 4.0) and extreme alkaline (pH 9.0 and pH 10.0) conditions, compared to YBT-1518 and BMB1684 strains (Fig. 7E). These data suggest that the CRISPR-Cas system negatively impacts bacteria tolerance to extreme stresses.

To confirm the above observations, we introduced the cloned VD115 CRISPR/Cas system into another model *B. thuringiensis* strain BMB171 that lacks CRISPR/Cas system. Similar to BMB1685 strain that was transformed with the complete subtype I-C system, (Fig. 7), the introduction of a functional subtype I-C system in BMB171 strain resulted in reduced bacteria growth, sporulation rate, biofilm formation ability, and adaptability to various stress, including acid, alkaline, and high salts tolerance (Fig. S10). We also confirmed by qPCR analysis that the introduced of CRISPR-Cas system leading to the observed phenotypes do not depend on the cost of wide defects on gene expression by analyzing the expression of two widely used reference genes in *B. cereus* group strains, *gatB* and *rpoA* [[51](#_ENREF_51)] (detailed information was shown in the Supplementary Text, Fig. S11A). showing that these two housekeeping genes have similar expression level at the same growth stage in wildtype strain YBT1518, and recombinant strain BMB1685 (Fig. S11A). Thus, we concluded that the active CRISPR-Cas system increased the fitness cost of host, and displays negative impact on host adaptation.

**Active CRISPR-Cas system interferes with *B. thuringiensis* pathogenicity**

*B. thuringiensis* YBT-1518 is a nematicidal strain which can complete its life cycle in *C. elegans* after colonization of the worm intestine [[40](#_ENREF_40)]. To determine the effect of CRISPR-Cas system on the adaptation of YBT-1518 in its host, we analyzed the colonization capacity of BMB1685 strain expressing CRISPR Cas I-C system in *C. elegans* after 24 and 48 h post-infections. The results showed that the number of *B. thuringiensis* colonies obtained from worms infected with of BMB1685 was significantly reduced in comparison with worms infected with either YBT-1518 or BMB1684 strains (Fig. 7F). Colonization of *B. thuringiensis* in the nematode intestine leads to nematode mortality. To analyze the effect of CRISPR-Cas system on the pathogenicity of *B. thuringiensis* BMB1685, we determined the survival of *C. elegans* fed with the different *B. thuringiensis* strains. The results show that nematodes fed with BMB1685 have higher survival rates than that those fed with YBT-1518 and BMB1684 strains in the mortality assays of *C. elegans* (Fig. 7G).

**Discussion**

The main conclusion of our work is that the CRISPR-Cas systems are barriers to HGT within *B. cereus* group of bacteria correlating with wider environmental distribution. In accordance, strains harboring active CRISPR-Cas systems have limited environmental distributions (Fig. 3 and Fig. 4), less MGEs and lower number of unique genes in their genomes (Fig. 5), as compared to strains without active CRISPR-Cas systems.

We found that *B. cereus* group harbor only CRISPR-Cas Systems subtypes I-C and I-B, which agrees with the trend observed in all bacteria genome sequences characterized showing that type I systems are the most frequently found in bacteria (60%) (6). It is interesting to note that it was proposed that subtype I-C seems to be a derivative of subtype I-B [[26](#_ENREF_26)]. Our data show that the distribution rate of CRISPR-Cas systems is largely different among ecotypes of *B. cereus* group. Also, that the distribution seems to depend on the environmental niches of certain strains (Fig. 3). For example, *Bacillus* strains with complete CRISPR-Cas systems showed reduced distribution in different ecological niches (Fig 4A), while many of the *B. thuringiensis*, *B. cereus* and *B. cytotoxicus* strains, which can be isolated from more diverse ecological niches, have incomplete CRISPER-Cas system or lack these systems (Fig 4). CRISPR-Cas systems are also widely found in 87% Archaea and 45% bacterial genomes, most of which are bacterial pathogens (<http://crispr.i2bc.paris-saclay.fr/>) and are found in specific environmental niches. Thus, we proposed that Archaea and bacterial pathogens from single environmental niches may have larger distribution of complete functional CRISPR-Cas systems, while Archaea or bacteria from open or diverse environments may have less active systems. A reasonable explanation is that inactivation of the CRISPR-Cas systems allowed higher frequency of HGT to obtain selective genetic traits for better adaptability to diverse environments. This proposition is in agreement with the proposal that pangenomes resulted from an adaptive evolution, where HGT positively increases the possibility to acquire advantageous genes allowing prokaryotic species to migrate to new niches [[52](#_ENREF_52)]. However, we cannot ignore the defense role of CRISPR-Cas systems in protecting the genome from phages and transposons invasion explaining that most spacer targets actually come from integrated elements such as phages and transposons as found in this work (Dataset 2 and Fig. S1) and previously reported [[53](#_ENREF_53)]. The fact that the CRISPR–Cas systems in most of *B. cereus* group strains are often lost or inactivated (Fig. 1 and Fig. 2), together with the data that strains harboring active systems have less MGEs (Fig. 5), strongly suggest that inactivation of CRISPR-Cas systems should be an evolution trade-off between individual self-protection and better adaptability of the *B. cereus* group population.

Another important observation was that the inactivation pathways of CRISPR-Cas systems in *B. cereus* group mainly involves the replacement or deletion of *cas* genes that are responsible for acquiring new spacers (Fig. 2). The spacer acquisition is important for the immune function of CRISPR-Cas systems, and the spacer diversity in CRISPR array reflects the interaction frequency between the bacteria and the invasive nucleic acids [[1](#_ENREF_1)]. In accordance, more spacers and higher diversity of spacer compositions was found in strains with intact CRISPER-Cas systems compared to those lacking CRISPER-Cas systems or containing the incomplete ones (Fig. 2D). Losing the spacer acquisition ability but keeping other components may have some special biological significance for certain strains (164 of 260), since CRISPR-Cas systems have been shown to possess several other unexpected roles beyond its immune function [[10](#_ENREF_10)]. However, the factors that determined such inactivation pathways of CRISPR-Cas systems in *B. cereus* group, and the detailed mechanisms and significance involved remain unclear and need further studies. Other inactivation models of CRISPR-Cas systems include loss of nuclease components or repression of the expression of nucleases [[14](#_ENREF_14), [15](#_ENREF_15)] and attenuating the function by diverse anti-CRISPR proteins [[54](#_ENREF_54)]. In addition, loss of CRISPR-Cas elements through recombination has been reported [[55](#_ENREF_55)].

It has been shown that CRISPR-Cas systems limited antibiotic resistance in the pathogens *Klebsiella pneumoniae* [[56](#_ENREF_56)] and *E. coli* [[57](#_ENREF_57)]. Perhaps the benefit of acquiring new MGEs for better adaptation to new environments outweighed the costs of phage infection, and additional anti-phage systems may be present in these bacteria. BLASTP analysis and domain searching revealed that other anti-phages systems including restriction (R)-modification (M) systems, Druantia, and Gabija systems, etc. [[58-61](#_ENREF_58)], are widely distributed within *B. cereus* group strains (Table S11). These results suggests that inactivation of CRISPR-Cas systems resulted in enhanced HGT that could have detrimental effects due to enhanced infection with phages and transposons which may be overcome by the acquisition of additional and different anti-phage protection system (such as DISARM, Druantia, Gabija, Lamassu, Septu, Thoeris or Zorya-I systems). In accordance with this hypothesis, the number of such anti-phages systems is much higher in the strains that have incomplete CRISPR-Cas systems than those with a complete system (Fig. S12). These data may explain why the number of putative transposases, prophage elements, plasmids, and the number of unique genes per genome are significantly higher in the in strains with incomplete CRISPR-Cas than those strains lacking CRISPR-Cas (Fig. 5). Thus, deletion of CRISPR-Cas systems may have co-evolved with the acquisition of alternative immune systems. We indeed showed that the functional CRISPR-Cas system in BMB1685 strain reduces the adaptation of this transformant bacterium under both host infection and environmental conditions (Fig. 7). We have shown that spacers identified could target multiple chromosomal genes as self- targeting, intra-species targeting or inter-species targeting (Fig. S2 and Dataset 1). The self- targeting spacers may raise the issue of auto-immunity in the host. Thus, the observed effects in BMB1685 strain, may indicate activation of autoimmunity responses affecting the performance of BMB1685 on the different conditions analyzed. We do not know which genes were target in these assays and further studies will help to better understand the disadvantages of expressing such CRISPR-Cas system in BMB1685. While the inter-species targeting spacers has proposed may limit inter-species HGT [[42](#_ENREF_42)]. Therefore, the large numbers of intra-species targeting may limit the HGT among strains in the *B. cereus* group. Thus, the autoimmunity responses and intra-species targeting may explain why most *B. cereus* group strains evolve to inactivate CRISPR-Cas systems. Our work puts forward a novel adaptive strategy employed by bacteria via inactivation of CRISPR-Cas systems by deletion of certain key components, reducing autoimmunity responses and facilitating the acquisition of potentially beneficial MGEs.

As pathogens for insects and nematodes, *B. thuringiensis* produces diverse virulence factors [[40](#_ENREF_40), [62-66](#_ENREF_62)]. Production of multiple virulence factors benefits the populations by facilitating host infection, that represent more resources. Comparative genome analysis performed with a highly insecticidal *B. thuringiensis* HD-1 strain [[67](#_ENREF_67)] as reference, revealed that *B. thuringiensis* strains with active CRISPR-Cas systems contain less virulence factors than strains without active CRISPR-Cas systems (Fig. S13). This information indicates that the CRISPR-Cas systems prevent the transfer of virulence genes within pathogens. The CRISPR-Cas systems in *S. pneumoniae* and *Streptococcus pyogenes* were also shown to prevent acquisition of virulence factors [[11](#_ENREF_11), [68](#_ENREF_68)]. Thus, the negative effect of CRISPR-Cas systems for pathogenicity and adaptability may be a common phenomenon, suggesting that the strategy of CRISPR-Cas systems inactivation could improve the adaptability of various bacterial pathogens to their hosts.

**Supplementary information**

Supplementary information is available at The ISME Journal Online.

**Acknowledgements**

We thank Dr. Didier Lereclus (INRA, Jouy-en-Josas, France) for providing plasmid pHT315. We also thank the *Caenorhabditis* Genetics Center for the worm strain. This work was supported by the National Key Research and Development Program of China (2017YFD0201201 and 2017YFD0200400); the National Natural Science Foundation of China (31670085 and 31770116), the Fundamental Research Funds for the Central Universities (2662016PY067), projects funded by China Postdoctoral Science Foundation (2017M622473 and 2019T120669) and Postdoctoral Science and Technology Activities in Hubei Province (Z43).

**Conflict of interest**

The authors disclose no conﬂicts of interest.

**References**

1. van der Oost J, Westra ER, Jackson RN, Wiedenheft B. Unravelling the structural and mechanistic basis of CRISPR-Cas systems. Nature Reviews Microbiology. 2014;12(7):479-92.

2. Barrangou R, Marraffini LA. CRISPR-Cas systems: prokaryotes upgrade to adaptive immunity. Molecular cell. 2014;54(2):234-44.

3. Barrangou R, Fremaux C, Deveau H, Richards M, Boyaval P, Moineau S, et al. CRISPR provides acquired resistance against viruses in prokaryotes. Science. 2007;315(5819):1709-12.

4. Marraffini LA, Sontheimer EJ. CRISPR interference limits horizontal gene transfer in staphylococci by targeting DNA. Science. 2008;322(5909):1843-5. doi: 10.1126/science.1165771. PubMed PMID: 19095942; PubMed Central PMCID: PMC2695655.

5. Makarova KS, Haft DH, Barrangou R, Brouns SJ, Charpentier E, Horvath P, et al. Evolution and classification of the CRISPR–Cas systems. Nature Reviews Microbiology. 2011;9(6):467-77.

6. Makarova KS, Wolf YI, Alkhnbashi OS, Costa F, Shah SA, Saunders SJ, et al. An updated evolutionary classification of CRISPR-Cas systems. Nat Rev Microbiol. 2015;13(11):722-36. doi: 10.1038/nrmicro3569. PubMed PMID: 26411297; PubMed Central PMCID: PMC5426118.

7. Shmakov S, Smargon A, Scott D, Cox D, Pyzocha N, Yan W, et al. Diversity and evolution of class 2 CRISPR-Cas systems. Nat Rev Microbiol. 2017;15(3):169-82. doi: 10.1038/nrmicro.2016.184. PubMed PMID: 28111461; PubMed Central PMCID: PMC5851899.

8. Zetsche B, Gootenberg JS, Abudayyeh OO, Slaymaker IM, Makarova KS, Essletzbichler P, et al. Cpf1 Is a Single RNA-Guided Endonuclease of a Class 2 CRISPR-Cas System. Cell. 2015;163(3):759-71. doi: 10.1016/j.cell.2015.09.038. PubMed PMID: WOS:000364828900027.

9. Barrangou R, Doudna JA. Applications of CRISPR technologies in research and beyond. Nat Biotechnol. 2016;34(9):933-41. doi: 10.1038/nbt.3659. PubMed PMID: 27606440.

10. Faure G, Makarova KS, Koonin EV. CRISPR-Cas: Complex Functional Networks and Multiple Roles beyond Adaptive Immunity. Journal of molecular biology. 2019;431(1):3-20. doi: 10.1016/j.jmb.2018.08.030. PubMed PMID: 30193985.

11. Bikard D, Hatoum-Aslan A, Mucida D, Marraffini LA. CRISPR interference can prevent natural transformation and virulence acquisition during in vivo bacterial infection. Cell Host Microbe. 2012;12(2):177-86. doi: 10.1016/j.chom.2012.06.003. PubMed PMID: 22901538.

12. Sampson TR, Saroj SD, Llewellyn AC, Tzeng YL, Weiss DS. A CRISPR/Cas system mediates bacterial innate immune evasion and virulence. Nature. 2013;497(7448):254-7. doi: 10.1038/nature12048. PubMed PMID: 23584588; PubMed Central PMCID: PMC3651764.

13. Heidrich N, Hagmann A, Bauriedl S, Vogel J, Schoen C. The CRISPR/Cas system in Neisseria meningitidis affects bacterial adhesion to human nasopharyngeal epithelial cells. RNA biology. 2019;16(4):390-6. doi: 10.1080/15476286.2018.1486660. PubMed PMID: WOS:000465256900003.

14. Palmer KL, Gilmore MS. Multidrug-resistant enterococci lack CRISPR-cas. MBio. 2010;1(4). doi: 10.1128/mBio.00227-10. PubMed PMID: 21060735; PubMed Central PMCID: PMC2975353.

15. Hullahalli K, Rodrigues M, Nguyen UT, Palmer K. An attenuated CRISPR-Cas system in *Enterococcus faecalis* permits DNA acquisition. mBio. 2018;9(3). doi: 10.1128/mBio.00414-18. PubMed PMID: 29717009; PubMed Central PMCID: PMC5930301.

16. Gophna U, Kristensen DM, Wolf YI, Popa O, Drevet C, Koonin EV. No evidence of inhibition of horizontal gene transfer by CRISPR-Cas on evolutionary timescales. ISME J. 2015;9(9):2021-7. Epub 2015/02/25. doi: 10.1038/ismej.2015.20. PubMed PMID: 25710183; PubMed Central PMCID: PMCPMC4542034.

17. Du Toit A. Viral infection: CRISPR-Cas enhances HGT by transduction. Nature reviews Microbiology. 2018;16(4):186. doi: 10.1038/nrmicro.2018.28. PubMed PMID: 29479076.

18. Watson BNJ, Staals RHJ, Fineran PC. CRISPR-Cas-mediated phage resistance enhances horizontal gene transfer by transduction. MBio. 2018;9(1). Epub 2018/02/15. doi: 10.1128/mBio.02406-17. PubMed PMID: 29440578; PubMed Central PMCID: PMCPMC5821089.

19. Varble A, Meaden S, Barrangou R, Westra ER, Marraffini LA. Recombination between phages and CRISPR-cas loci facilitates horizontal gene transfer in staphylococci. Nat Microbiol. 2019. doi: 10.1038/s41564-019-0400-2. PubMed PMID: 30886355.

20. Cohan FM. Bacterial species and speciation. Syst Biol. 2001;50(4):513-24. Epub 2002/07/16. doi: 10.1080/10635150118398. PubMed PMID: 12116650.

21. Schnepf E, Crickmore N, Van Rie J, Lereclus D, Baum J, Feitelson J, et al. Bacillus thuringiensis and its pesticidal crystal proteins. Microbiology and molecular biology reviews : MMBR. 1998;62(3):775-806. PubMed PMID: 9729609; PubMed Central PMCID: PMC98934.

22. Kolsto AB, Tourasse NJ, Okstad OA. What sets Bacillus anthracis apart from other Bacillus species? Annual review of microbiology. 2009;63:451-76. doi: 10.1146/annurev.micro.091208.073255. PubMed PMID: 19514852.

23. Kovac J, Miller RA, Carroll LM, Kent DJ, Jian J, Beno SM, et al. Production of hemolysin BL by *Bacillus cereus* group isolates of dairy origin is associated with whole-genome phylogenetic clade. BMC genomics. 2016;17:581. doi: 10.1186/s12864-016-2883-z. PubMed PMID: 27507015; PubMed Central PMCID: PMC4979109.

24. Yang J, Wang L, Ji X, Feng Y, Li X, Zou C, et al. Genomic and proteomic analyses of the fungus *Arthrobotrys oligospora* provide insights into nematode-trap formation. PLoS pathogens. 2011;7(9):e1002179. doi: 10.1371/journal.ppat.1002179. PubMed PMID: 21909256; PubMed Central PMCID: PMC3164635.

25. Grissa I, Vergnaud G, Pourcel C. CRISPRFinder: a web tool to identify clustered regularly interspaced short palindromic repeats. Nucleic acids research. 2007;35(Web Server issue):W52-7. doi: 10.1093/nar/gkm360. PubMed PMID: 17537822; PubMed Central PMCID: PMC1933234.

26. Makarova KS, Wolf YI, Koonin EV. The basic building blocks and evolution of CRISPR-CAS systems. Biochemical Society transactions. 2013;41(6):1392-400. doi: 10.1042/BST20130038. PubMed PMID: 24256226; PubMed Central PMCID: PMC5898231.

27. Page AJ, Cummins CA, Hunt M, Wong VK, Reuter S, Holden MT, et al. Roary: rapid large-scale prokaryote pan genome analysis. Bioinformatics. 2015;31(22):3691-3. Epub 2015/07/23. doi: 10.1093/bioinformatics/btv421. PubMed PMID: 26198102; PubMed Central PMCID: PMCPMC4817141.

28. Edgar RC. MUSCLE: multiple sequence alignment with high accuracy and high throughput. Nucleic acids research. 2004;32(5):1792-7. doi: 10.1093/nar/gkh340. PubMed PMID: 15034147; PubMed Central PMCID: PMC390337.

29. Price MN, Dehal PS, Arkin AP. FastTree: computing large minimum evolution trees with profiles instead of a distance matrix. Mol Biol Evol. 2009;26(7):1641-50. doi: 10.1093/molbev/msp077. PubMed PMID: 19377059; PubMed Central PMCID: PMC2693737.

30. Riadi G, Medina-Moenne C, Holmes DS. TnpPred: a web service for the robust prediction of prokaryotic transposases. Comparative and functional genomics. 2012;2012:678761. doi: 10.1155/2012/678761. PubMed PMID: 23251097; PubMed Central PMCID: PMC3506888.

31. Potter SC, Luciani A, Eddy SR, Park Y, Lopez R, Finn RD. HMMER web server: 2018 update. Nucleic acids research. 2018;46(W1):W200-W4. doi: 10.1093/nar/gky448. PubMed PMID: 29905871; PubMed Central PMCID: PMC6030962.

32. Zheng J, Peng D, Ruan L, Sun M. Evolution and dynamics of megaplasmids with genome sizes larger than 100 kb in the *Bacillus cereus* group. BMC Evol Biol. 2013;13:262. Epub 2013/12/04. doi: 10.1186/1471-2148-13-262. PubMed PMID: 24295128; PubMed Central PMCID: PMCPMC4219350.

33. Punta M, Coggill PC, Eberhardt RY, Mistry J, Tate J, Boursnell C, et al. The Pfam protein families database. Nucleic acids research. 2012;40(Database issue):D290-301. doi: 10.1093/nar/gkr1065. PubMed PMID: 22127870; PubMed Central PMCID: PMCPMC3245129.

34. Arndt D, Grant JR, Marcu A, Sajed T, Pon A, Liang Y, et al. PHASTER: a better, faster version of the PHAST phage search tool. Nucleic acids research. 2016;44(W1):W16-21. doi: 10.1093/nar/gkw387. PubMed PMID: 27141966; PubMed Central PMCID: PMCPMC4987931.

35. Meric G, Mageiros L, Pascoe B, Woodcock DJ, Mourkas E, Lamble S, et al. Lineage-specific plasmid acquisition and the evolution of specialized pathogens in *Bacillus thuringiensis* and the *Bacillus cereus* group. Mol Ecol. 2018;27(7):1524-40. doi: 10.1111/mec.14546. PubMed PMID: 29509989; PubMed Central PMCID: PMCPMC5947300.

36. Peng D, Luo Y, Guo S, Zeng H, Ju S, Yu Z, et al. Elaboration of an electroporation protocol for large plasmids and wild-type strains of Bacillus thuringiensis. Journal of applied microbiology. 2009;106(6):1849-58. doi: 10.1111/j.1365-2672.2009.04151.x. PubMed PMID: 19291242.

37. Stiernagle T. Maintenance of C. elegans. WormBook : the online review of C elegans biology. 2006:1-11. doi: 10.1895/wormbook.1.101.1. PubMed PMID: 18050451; PubMed Central PMCID: PMC4781397.

38. O'Toole GA. Microtiter dish biofilm formation assay. J Vis Exp. 2011;(47). Epub 2011/02/11. doi: 10.3791/2437. PubMed PMID: 21307833; PubMed Central PMCID: PMCPMC3182663.

39. Wang M, Du J, Zhang D, Li X, Zhao J. Modification of Different Pulps by Homologous Overexpression Alkali-Tolerant Endoglucanase in Bacillus subtilis Y106. Sci Rep. 2017;7(1):3321. Epub 2017/06/14. doi: 10.1038/s41598-017-03215-9. PubMed PMID: 28607421; PubMed Central PMCID: PMCPMC5468302.

40. Peng D, Lin J, Huang Q, Zheng W, Liu G, Zheng J, et al. A novel metalloproteinase virulence factor is involved in Bacillus thuringiensis pathogenesis in nematodes and insects. Environ Microbiol. 2016;18(3):846-62. doi: 10.1111/1462-2920.13069. PubMed PMID: 26995589.

41. Stern A, Keren L, Wurtzel O, Amitai G, Sorek R. Self-targeting by CRISPR: gene regulation or autoimmunity? Trends Genet. 2010;26(8):335-40. doi: 10.1016/j.tig.2010.05.008. PubMed PMID: WOS:000280903800001.

42. Turgeman-Grott I, Joseph S, Marton S, Eizenshtein K, Naor A, Soucy SM, et al. Pervasive acquisition of CRISPR memory driven by inter-species mating of archaea can limit gene transfer and influence speciation. Nat Microbiol. 2019;4(1):177-+. doi: 10.1038/s41564-018-0302-8. PubMed PMID: WOS:000453056100022.

43. Zheng J, Gao Q, Liu L, Liu H, Wang Y, Peng D, et al. Comparative Genomics of Bacillus thuringiensis Reveals a Path to Specialized Exploitation of Multiple Invertebrate Hosts. mBio. 2017;8(4). doi: 10.1128/mBio.00822-17. PubMed PMID: 28790205; PubMed Central PMCID: PMC5550751.

44. Ceuppens S, Boon N, Uyttendaele M. Diversity of *Bacillus cereus* group strains is reflected in their broad range of pathogenicity and diverse ecological lifestyles. FEMS microbiology ecology. 2013;84(3):433-50. doi: 10.1111/1574-6941.12110. PubMed PMID: 23488744.

45. Raymond B, Bonsall MB. Cooperation and the evolutionary ecology of bacterial virulence: the *Bacillus cereus* group as a novel study system. Bioessays. 2013;35(8):706-16. doi: 10.1002/bies.201300028. PubMed PMID: 23702950.

46. McDaniel LD, Young E, Delaney J, Ruhnau F, Ritchie KB, Paul JH. High frequency of horizontal gene transfer in the oceans. Science. 2010;330(6000):50. Epub 2010/10/12. doi: 10.1126/science.1192243. PubMed PMID: 20929803.

47. Bergstrom CT, Lipsitch M, Levin BR. Natural selection, infectious transfer and the existence conditions for bacterial plasmids. Genetics. 2000;155(4):1505-19. PubMed PMID: 10924453; PubMed Central PMCID: PMCPMC1461221.

48. Rancurel C, Legrand L, Danchin EGJ. Alienness: rapid detection of candidate horizontal gene transfers across the tree of life. Genes (Basel). 2017;8(10). doi: 10.3390/genes8100248. PubMed PMID: 28961181; PubMed Central PMCID: PMCPMC5664098.

49. Zhang F, Peng D, Cheng C, Zhou W, Ju S, Wan D, et al. *Bacillus thuringiensis* crystal protein Cry6Aa triggers *Caenorhabditis elegans* necrosis pathway mediated by aspartic protease (ASP-1). PLoS pathogens. 2016;12(1):e1005389. Epub 2016/01/23. doi: 10.1371/journal.ppat.1005389. PubMed PMID: 26795495; PubMed Central PMCID: PMCPMC4721865.

50. Burmolle M, Ren D, Bjarnsholt T, Sorensen SJ. Interactions in multispecies biofilms: do they actually matter? Trends in microbiology. 2014;22(2):84-91. Epub 2014/01/21. doi: 10.1016/j.tim.2013.12.004. PubMed PMID: 24440178.

51. Reiter L, Kolsto AB, Piehler AP. Reference genes for quantitative, reverse-transcription PCR in Bacillus cereus group strains throughout the bacterial life cycle. J Microbiol Methods. 2011;86(2):210-7. Epub 2011/05/31. doi: 10.1016/j.mimet.2011.05.006. PubMed PMID: 21620905.

52. McInerney JO, McNally A, O'Connell MJ. Why prokaryotes have pangenomes. Nat Microbiol. 2017;2(4). doi: ARTN 17040

10.1038/nmicrobiol.2017.40. PubMed PMID: WOS:000398177800019.

53. Shmakov SA, Sitnik V, Makarova KS, Wolf YI, Severinov KV, Koonin EV. The CRISPR Spacer Space Is Dominated by Sequences from Species-Specific Mobilomes. Mbio. 2017;8(5). doi: ARTN e01397-17

10.1128/mBio.01397-17. PubMed PMID: WOS:000416271100059.

54. Pawluk A, Davidson AR, Maxwell KL. Anti-CRISPR: discovery, mechanism and function. Nature reviews Microbiology. 2018;16(1):12-7. doi: 10.1038/nrmicro.2017.120. PubMed PMID: 29062071.

55. Horvath P, Romero DA, Coute-Monvoisin AC, Richards M, Deveau H, Moineau S, et al. Diversity, activity, and evolution of CRISPR loci in Streptococcus thermophilus. Journal of bacteriology. 2008;190(4):1401-12. doi: 10.1128/JB.01415-07. PubMed PMID: 18065539; PubMed Central PMCID: PMC2238196.

56. Li HY, Kao CY, Lin WH, Zheng PX, Yan JJ, Wang MC, et al. Characterization of CRISPR-Cas systems in clinical *Klebsiella pneumoniae* isolates uncovers its potential association with antibiotic susceptibility. Front Microbiol. 2018;9:1595. Epub 2018/08/01. doi: 10.3389/fmicb.2018.01595. PubMed PMID: 30061876; PubMed Central PMCID: PMCPMC6054925.

57. Long J, Xu Y, Ou L, Yang H, Xi Y, Chen S, et al. Polymorphism of Type I-F CRISPR/Cas system in *Escherichia coli* of phylogenetic group B2 and its application in genotyping. Infect Genet Evol. 2019;74:103916. Epub 2019/06/14. doi: 10.1016/j.meegid.2019.103916. PubMed PMID: 31195154.

58. Goldfarb T, Sberro H, Weinstock E, Cohen O, Doron S, Charpak-Amikam Y, et al. BREX is a novel phage resistance system widespread in microbial genomes. The EMBO journal. 2015;34(2):169-83. doi: 10.15252/embj.201489455. PubMed PMID: 25452498; PubMed Central PMCID: PMCPMC4337064.

59. Ofir G, Melamed S, Sberro H, Mukamel Z, Silverman S, Yaakov G, et al. DISARM is a widespread bacterial defence system with broad anti-phage activities. Nat Microbiol. 2018;3(1):90-8. doi: 10.1038/s41564-017-0051-0. PubMed PMID: 29085076; PubMed Central PMCID: PMCPMC5739279.

60. Doron S, Melamed S, Ofir G, Leavitt A, Lopatina A, Keren M, et al. Systematic discovery of antiphage defense systems in the microbial pangenome. Science. 2018;359(6379). doi: 10.1126/science.aar4120. PubMed PMID: 29371424.

61. Roberts RJ, Belfort M, Bestor T, Bhagwat AS, Bickle TA, Bitinaite J, et al. A nomenclature for restriction enzymes, DNA methyltransferases, homing endonucleases and their genes. Nucleic acids research. 2003;31(7):1805-12. PubMed PMID: 12654995; PubMed Central PMCID: PMCPMC152790.

62. Luo X, Chen L, Huang Q, Zheng J, Zhou W, Peng D, et al. Bacillus thuringiensis metalloproteinase Bmp1 functions as a nematicidal virulence factor. Applied and environmental microbiology. 2013;79(2):460-8. doi: 10.1128/AEM.02551-12. PubMed PMID: 23124228; PubMed Central PMCID: PMC3553750.

63. Ruan L, Wang H, Cai G, Peng D, Zhou H, Zheng J, et al. A two-domain protein triggers heat shock pathway and necrosis pathway both in model plant and nematode. Environ Microbiol. 2015;17(11):4547-65. doi: 10.1111/1462-2920.12968. PubMed PMID: 26147248.

64. Liu XY, Ruan LF, Hu ZF, Peng DH, Cao SY, Yu ZN, et al. Genome-wide screening reveals the genetic determinants of an antibiotic insecticide in *Bacillus thuringiensis*. The Journal of biological chemistry. 2010;285(50):39191-200. doi: 10.1074/jbc.M110.148387. PubMed PMID: 20864531; PubMed Central PMCID: PMC2998090.

65. Du C, Cao S, Shi X, Nie X, Zheng J, Deng Y, et al. Genetic and biochemical characterization of a gene operon for trans-aconitic acid, a novel nematicide from *Bacillus thuringiensis*. The Journal of biological chemistry. 2017;292(8):3517-30. doi: 10.1074/jbc.M116.762666. PubMed PMID: 28087696; PubMed Central PMCID: PMC5336182.

66. Zhao C, Luo Y, Song C, Liu Z, Chen S, Yu Z, et al. Identification of three Zwittermicin A biosynthesis-related genes from *Bacillus thuringiensis* subsp. *kurstaki* strain YBT-1520. Archives of microbiology. 2007;187(4):313-9. doi: 10.1007/s00203-006-0196-3. PubMed PMID: 17225146.

67. Zhu L, Peng D, Wang Y, Ye W, Zheng J, Zhao C, et al. Genomic and transcriptomic insights into the efficient entomopathogenicity of *Bacillus thuringiensis*. Scientific reports. 2015;5:14129. doi: 10.1038/srep14129. PubMed PMID: 26411888; PubMed Central PMCID: PMCPMC4585936.

68. Deltcheva E, Chylinski K, Sharma CM, Gonzales K, Chao Y, Pirzada ZA, et al. CRISPR RNA maturation by trans-encoded small RNA and host factor RNase III. Nature. 2011;471(7340):602-7. doi: 10.1038/nature09886. PubMed PMID: 21455174; PubMed Central PMCID: PMCPMC3070239.

**Tables**

**Table 1. Distribution of the CRISPR-Cas system types among *B. cereus* group members.**

| **Taxonomic group** | **Genomes analyzed** | **Type I system** | **Type II system** | **Type III system** | **Type IV system** | **Type V system** | **Type VI system** | **Occurrence of type I system** |
| --- | --- | --- | --- | --- | --- | --- | --- | --- |
| *B. anthracis* | 188 | 1 (0+1) | 0 | 0 | 0 | 0 | 0 | 0.5% |
| *B. cereus* | 862 | 75 (22+53) | 0 | 0 | 0 | 0 | 0 | 8.7% |
| *B. cytotoxicus* | 14 | 12 (8+4) | 1 (0+1) | 0 | 0 | 0 | 0 | 85.7% |
| *B. mycoides* | 57 | 5 (1+4) | 0 | 0 | 0 | 0 | 0 | 8.8% |
| *B. pseudomycoides* | 104 | 29 (29+0) | 0 | 0 | 0 | 0 | 0 | 27.9% |
| *B. thuringiensis* | 445 | 116 (14+102) | 0 | 0 | 0 | 0 | 0 | 26.1% |
| *B. toyonensis* | 201 | 22 (2+20) | 0 | 0 | 0 | 0 | 0 | 10.9% |
| **Total** | **1,871** | **260 (76+184)** | **1 (0+1)** | **0** | **0** | **0** | **0** | **13.9%** |

Note: The labels in red and blue in column of “Type I system” represent number of complete and incomplete CRISPR-Cas systems, respectively.

**Figure Legends:**

**Fig. 1. Analysis of CRISPR-Cas systems in the *B. cereus* group.** **(A)** Genetic organization of type I CRISPR-Cas systems found in *B. cereus* group. Only the representative complete subtypes are shown. **(B)** Proportion of *B. cereus* group strains with complete or incomplete CRISPR-Cas systems. Subtypes I-B, I-B-like and I-C are complete systems, and all systems, which lack any *cas* genes compared to the complete systems are defined as incomplete.

**Fig. 2.** **Genetic inactivation of CRISPR-Cas system commonly occurs in *B. cereus* group.** **(A)** Comparison of the genetic organization of variants of type I CRISPR-Cas systems. All the sequences were retrieved from NCBI and their accession numbers are listed in Table S1. Only representative strains are shown. **(B)** The maximum-likelihood (ML) phylogenetic tree based on the sequence of Cas7 protein was constructed by FastTree. The strains with intact or variants of subtypes I-C systems are shown in red branches, while the intact or variants of subtypes I-B systems are shown in blue branches. Nodes supported with a bootstrap values ≥ 50% are indicated with a black dot. Ecotypes names of *B. cereus* group are abbreviated as follows: Ban for *B. anthracis*, Bce for *B. cereus*, Bcy for *B. cytotoxicus*, Bmy for *B. mycoides*, Bps for *B. pseudomycoides*, Bth for *B. thuringiensis*, and Bto for *B. toyonensis*. The numbers, such as GCA_002560815.1 and PFE79409.1 are the GenBank assembly accessions of genomes and GenBank accessions of Cas7 proteins, respectively. **(C)** Proposed inactivation pathways of type I CRISPR-Cas system in *B. cereus* group. The predicted inactivation pathways for subtypes I-C or subtypes I-B variants are shown. **(D)** Differences of spacer types among strains with variants of CRISPR-Cas system in the *B. cereus* group. The spacers of variant I-C d3 were excluded because only one strain contains this variant. All the spacers are listed in Dataset 1.

**Fig. 3. The distribution of** ***B. cereus* group strains with active- or inactive-** **CRISPR-Cas systems on population structure.** The ML tree was constructed by FastTree based on the protein sequences of single-copy core genes. Bootstrap support values were calculated from 1,000 replicates. Strain names were abbreviated as described in Figure 2. The background colors of the labels represent the clades: yellow, green, fuchsia, light green, olive and dark green for clades 1, 2, 3, 4, 5 and 6, respectively. The colors of labels represent states of CRISPR-Cas system: red, blue and black for complete, incomplete and none, respectively. Colors of the circle next to the labels represent the bacterial niches or hosts as follow: olive drab for earthworm Haplotaxida, peach puff for fish, indigo for Coleoptera, chocolate for Diptera, sienna for Homoptera, olive for Lepidoptera, coral for mouse, dark violet for nematode, dark orchid for nematode and insect, pink for human, lavender blush for human excrement, cyan for plant, brown yellow for plant core, green for plant leaf, light yellow for plant rhizosphere, violet for plant root, blue for soil, purple for food, fire brick for industrial product, navy for waste water, and white for unknown information. Inserts show column charts showing total numbers of the complete (red bars) and incomplete systems (blue bars) in each Clade.

**Fig. 4. The environmental niches distribution of *B. cereus* group strains with active- or inactive- CRISPR-Cas systems.** **(A)** Total number of strains adapted to the different main types of ecological niches. The strains are grouped by complete, incomplete, and no type I CRISPR-Cas systems. **(B)** Comparison of the ratio of occupied ecological niches for strains with complete, incomplete or no CRISPR-Cas systems. The data are shown as proportion of the total niches colonized by all strains in each group.

**Fig. 5. Distribution of mobile genetic elements and unique genes among selected *B. cereus* group strains.** The MGEs including transposases **(A)**, plasmid replication essential proteins **(B)**, and prophage proteins **(C)** contents among selected strains with complete, incomplete or no CRISPR-Cas systems are shown. The contents were compared by calculating the numbers of each kind of MGEs per genome of each group. **(D)** The comparison of unique gene diversity value among *B. cereus* group strains. Unique gene diversity was compared by calculating the rate of unique genes per genome for all the selected genomes. The significance of the differences among samples was evaluated using two sample Wilcoxon-Mann-Whitney test at, *p* < 0.0001 (****) *p* < 0.001 (***), *p* < 0.01 (**) and *p* < 0.05 (*).

**Fig. 6. Functional analysis of the subtype I-C CRISPR-Cas system from *B. cereus* strain VD115. (A)** The genetic organization of *cas* operon in subtype I-C CRISPR-Cas system loci from *B. cereus* VD115. **(B)** Plasmids derivatives of pHT-304 containing synthetic VD115 spacer 1 accompanied by 5’-TTN or 5’-NNN PAMs (TGC, TAC, GTC and ATC) were transformed into *B. cereus* VD115 strain and the transformation efficiency of plasmids was analyzed and showed as the number of CFU/ml. The immunity assays of subtype I-C CRISPR-Cas locus in *B. cereus* VD115 **(C)** and in *B. thuringiensis* BMB1685 **(D)** strains. The immunity was assessed via transformation of plasmids containing spacers flanked by a 5’-TTC PAM site in both strains. The transformation was conducted via electroporation, and the results were expressed as the number of CFU/ml. The pHT-304 vector was used as negative control in all assays. **(E)** T-A cloning and sequencing demonstrated the deletion region of *cry6Aa* gene*.* A loss of 198 bps in the edited *cry6A* (∆*cry6Aa*) is shown, compared to the wild type *cry6Aa* gene in BMB1685 strain. **(F)** The Cry6Aa protein production in BMB1685 strain and in selected *cry6Aa* deletion mutants was analyzed by SDS-PAGE. The red arrow points to the band of the complete Cry6Aa protein.

**Fig. 7**. **Impact of active CRISPR-Cas system on the adaptation to different environmental stresses and into its nematode host. (A)** Growth curve of *B. thuringiensis* transformed with subtype I-C CRISPR-Cas system (BMB1685) or same *B. thuringiensis* strain without transformation (YBT-1518) or transformed with empty vector (BMB1684). The optical absorbance value at 600 nm of these *B. thuringiensis* strains grown in LB medium was analyzed successively from 0 to 54 h. **(B)** Sporulation rate of bacteria. Sporulation rates were calculated by the CFU of spore-containing aliquots heated at 80°C for 10 min divide by CFU of the aliquots that were kept on ice before growing in LB-agar plates for 24 h at 28°C. **(C)** Biofilm formation ability of bacteria. The data are expressed as the absorbance at 595 nm of wells treated with different *B. thuringiensis* strains after staining**. (D)** Salt tolerance of bacteria. The data are expressed as survival rate of the different *B. thuringiensis* strains exposed to high-salt environments containing 2%, 4% and 6% NaCl. **(E)** pH tolerance of bacteria. The data are expressed as survival rate of *B. thuringiensis* strains exposed to different pH environments. In all assays, BMB1684 strain containing empty pBAC44 vector was used as control**. (F)** Colonization of the different *B. thuringiensis* strains in *C. elegans*. A total of 10^3^ spores of each *B. thuringiensis* strain were added to 30-50 L4 stage N2 nematodes as the only food source. The colonies of *B. thuringiensis* were counted after analyzing the samples isolated from infected worms after 24 and 48 h. The BMB1684 strain containing empty pBAC44 vector was used as control. **(G)** Life-span assay of *C. elegans* feeding with *B. thuringiensis* with or without of subtype I-C CRISPR-Cas system. The survival rates of L4 stage N2 worms (50-100 for each well) fed with spore/crystal mixture of the different *B. thuringiensis* strains were calculated after different incubation times. The BMB1684 strain and standard food *E. coli* OP50 were used as controls. The error bars represent the standard deviations from the mean values of three independent experiments. The differences of recombinant strains against YBT-1518 wild type was evaluated using two-sample *t*-test. A double asterisk indicates *p* < 0.01, single asterisk indicates *p* < 0.05, lacking of any symbol and ND indicates no significant difference.
