# Supplementary material for: The CRISPR-Cas systems were selectively inactivated during evolution of Bacillus cereus group for adaptation to diverse environments
Source: ISME J. 2020 Mar 4;14(6):1479–93. doi: 10.1038/s41396-020-0623-5 (PMC7242445; doi:10.1038/s41396-020-0623-5)
Supplement: Supplementary file 1 — Supplementary materials [file 41396_2020_623_MOESM1_ESM.docx]

**Supplementary Material for**

**“The CRISPR-Cas systems were selectively inactivated during evolution of *Bacillus cereus* group for adaptation to diverse environments”**

**Running title:** Inactivation of CRISPR-Cas systems in Bc group

Ziqiang Zheng^1^, Yulan Zhang^1^, Zhiyu Liu^1^, Zhaoxia Dong^1^, Chuanshuai Xie^1^, Alejandra Bravo^2^, Mario Soberón^2^, Jacques Mahillon^3^, Ming Sun^1^*, and Donghai Peng^1^*

^1^State Key Laboratory of Agricultural Microbiology, College of Life Science and Technology, Huazhong Agricultural University, Wuhan 430070, Hubei, China

^2^Instituto de Biotecnología, Universidad Nacional Autónoma de México, Apdo. postal 510-3, Cuernavaca, 62250 Morelos, Mexico.

^3^Laboratory of Food and Environmental Microbiology, Earth and Life Institute, UCLouvain, Croix du Sud, 2 - L7.05.12, B-1348 Louvain-la-Neuve, Belgium

*Corresponding authors: E-mail: D.H.P. [donghaipeng@mail.hzau.edu.cn](mailto:donghaipeng@mail.hzau.edu.cn), or M.S. m98sun@mail.hzau.edu.cn; TEL: 86-27-87283455; FAX: 86-27-87280670.

**Supplementary Methods**

**Cloning of an intact subtype I-C CRISPR-Cas system from *B. cereus* VD115.**

The DNA fragment containing the intact subtype I-C CRISPR-Cas system locus of *B. cereus* VD115 was amplified with the primers listed in Table S9. The VD115-1S and VD115-1A primers were used to amplify F1 fragment, VD115-2S and VD115-2A primers were used to amplify F2 fragment and VD115-3S and VD115-3A primers were used for F3 fragment. The F1, F2 and F3 fragments were digested with *Hpa*I, *Hpa*I/*Hin*dIII and *Hin*dIII, respectively, ligated and cloned into *Bam*HI site of pBAC44 vector [1], resulting in plasmid pBMB1685. The construction procedure of pBMB1685 is depicted in Fig. S6. Plasmid pBMB1685 was then electroporated [2] into *B. thuringiensis* YBT-1518 resulting recombinant BMB1685.

**Plasmid transformation assays.**

Shuttle vector pHT-304 [3] was used to construct plasmids containing different spacers. These plasmids were transformed into the *B. cereus* VD115 or *B. thuringiensis* recombinant BMB1685 by electroporation [2]. The number of transformants were counted after 12 h incubation at 28°C in solid LB-agar medium. Strains VD115 and BMB1685 transformed with pHT-304 were used as controls.

**Supplementary Texts:**

**Functional characterization of an intact subtype I-C system from *B. cereus* VD115**

The Cas protein sequences in this system showed from 56 to 76% sequence identity against that of known subtype I-C system previously described in *Bacillus halodurans* C-125 [4] (Fig. S5). The expression of all *cas* genes was observed at 3, 6, 9 and 12 h of growth, suggesting that VD115 CRISPR-Cas system maybe functional (Fig. S7). Five protospacers of this system were identified from NCBI nucleic acid database, which were accompanied by 5’-TTC or 5’-TTT (Table S12), indicating the PAM sites of VD115 subtype I-C system is 5’-TTN.

The whole CRISPR-Cas locus of VD115 was then cloned into pBAC44 vector and transformed into *B. thuringiensis* YBT-1518, which contains no CRISPR-Cas elements, to generate the recombinant stain BMB1685 (Fig. S6). As control, the pBAC44 vector was transformed into YBT-1518 resulting in control recombinant BMB1684 strain.

For analyzing the genome editing function of the cloned subtype I-C system in strain BMB1685 a plasmid containing a small guide RNA (sgRNA) for *cry6Aa* gene was constructed. The sgRNA coding sequence, containing two repeats separated by a spacer from *cry6Aa* (Table S9), was synthesized and integrated into the downstream a P_kan_ promoter of pHT-315 vector. A sequence that comprises the upstream and downstream of *cry6Aa* gene was designed and followed the sgRNA for homologous recombination with *cry6Aa* (Fig. S8A). The plasmid was then introduced into BMB1685 and analyzed as stated in the text. Sixty-nine colonies were tested by PCR, and 98.5% of the colonies displayed a smaller band (568 bp) in agarose gel electrophoresis that represents the deletion in *cry6Aa* gene (Fig. S8B).

**The introduced of CRISPR-Cas system do not bring the cost of expression for Bacteria**

When transferring a cloned active CRISPR-Cas system to a strain that did not harbor such systems, the introduced system may be incompatible with the new host's RNA biology. To confirm this hypnosis, we analyzed the expression of two reference genes in *B. cereus* group strains, *gatB* and *rpoA*, by qPCR. When set as control genes for each other, we found these two housekeeping genes showed similar expression level at the same growth stage in wildtype strain YBT1518, and recombinant stains BMB1684 or BMB1685 (Fig. S11A). These data suggested that the introduced of an active CRISPR-Cas system do not bring cost of basic gene expression for host strain. Similarly, we repeated these experiments in another strain, and introduced the cloned VD115 CRISPR/Cas system into a model *B. thuringiensis* strain BMB171 that lacks any CRISPR/Cas system, resulting a recombinant strain BMB4027. When set as control genes for each other, the expression of *gatB* and *rpoA* genes in strains BMB171, BMB1682 (vector control), and BMB4027 showed similar expression level at the same growth stage (Fig. S11B). These phenotypes are very similar to observations when introduced the cloned VD115 CRISPR/Cas system into *B. thuringiensis* strain YBT1518. What’s more, we have showed that there are no growth differences in the mid-log growth stage among strains based on YBT1518 (YBT1518, BMB1684, and BMB1685) (Fig. 7A) or strains based on BMB171 (Fig. S10A). Take these data together, we concluded that the introduced of CRISPR-Cas system leading to the observed phenotypes do not depend on the cost of gene expression.

**TXT S1: Amino sequences of plasmid essential replication proteins.**

These plasmid essential replication proteins were retrieved from GenBank (<ftp://ftp.ncbi.nlm.nih.gov/genomes>) as previously described [5]. The candidate replication proteins identified by BLASTP were used to analyze the relationship between the CRISPR-Cas systems and mobile genetic elements (MGEs) in *Bacillus cereus s. l.* group. The detail information is in Table S5 and results are shown in Figure 4B.

**TXT S2: Domain file of tubz.hmm.**

Plasmid essential replication proteins with this domain were found out by hmmsearch and combined the candidates in TXT S1 for further analysis. The detail information is in Table S5 and results are shown in Fig. 4B.

**Supplementary Figures:**

**Fig. S1. Targets of CRISPR-Cas spacers in *B. cereus* group.** Targets of different spacers were identified by BLASTn in NCBI, details are listed in Dataset 2. The targets were classified into seven group, including targets that matched with sequence from plasmids, phages, chromosomes, plasmids and phages, plasmids and chromosomes, phages and chromosomes, and plasmids/phages and chromosomes.

**Fig. S2. The distribution of targets for the 1916 unique spacers identified from *B. cereus* group strains.** Self-targeting, spacers that match chromosomal loci in the same strain where the spacers come from; mobile DNA targeting, spacers that match mobile DNA loci including plasmids and phages; Inter-species targeting, spacers that match chromosomal loci in other species; Intra-species targeting, spacers that match chromosomal loci in other strains from the same species where the spacers were found. None identified, spacers that con not found any matched targets in the published database. The numbers at the top of each column mean the total number of unique spacers that were found.

**Fig. S3. The maximum-likelihood (ML) phylogenetic tree based on the sequence of Cas7 protein of all type I CRISPR-Cas systems in *B. cereus* group.** All the amino sequences of Cas7 proteins are from all type I CRISPR-Cas systems and the ML tree was constructed by FastTree [6]. Species names of *B. cereus s. l.* group are abbreviated as follows: Ban for *B. anthracis*, Bce for *B. cereus*, Bcy for *B. cytotoxicus*, Bmy for *B. mycoides*, Bps for *B. pseudomycoides*, Bth for *B. thuringiensis*, and Bto for *B. toyonensis*. The numbers, such as GCA_002560815.1 and PFE79409.1 are the GenBank assembly accessions of genomes and GenBank accessions of Cas7 proteins, respectively.

**(This figure was submitted directly as a single TIF file (designated as Fig. S3) because it contains a large amount of data that cannot be easily shown in this Word file).**

**Fig. S4. Spacers of representative intact or variant of subtype I-B and I-C CRISPR-Cas systems.** The spacers were verified with CRISPR-finder (<http://crispr.i2bc.paris-saclay.fr/>) [7]. All spacers in this study were designated with an order, such as S1314. The spacers with orange background indicate that the similarity among them is 100% in the spacer matrix.

**(This figure was submitted directly as a single TIF file (designated as Fig. S4) because it contains a large amount of data that cannot be easily shown in this Word file).**

**Fig. S5. Alignment between novel subtype I-C of *B. cereus* VD115 strain and known functional subtype I-C system of *B. halodurans* C-125 strain.** The open reading frames (ORFs) filled with different colors represent *cas* genes in the two CRISPR-Cas systems, and the percentages of similarity between these *cas* genes are shown in the figure. The rectangular blocks represent the CRISPR-Cas loci.

**Fig. S6. Cloning of subtype I-C system of *B. cereus* VD115 strain into pBAC44 vector.** The subtype I-C of VD115 strain was cloned by amplifying three fragments by PCR, followed by ligation, resulting in plasmid pBMB1685. The F3 fragment contains two CRISPR-Cas loci. The primers were listed in Table S8.

**Fig. S7. Transcription analysis of *cas* genes in *B. cereus* VD115 strain.** The RNA of VD115 strain was extracted with TranZolΜP Plus RNA Kit (TransGen Biotech Co. Ltd., Beijing, China), and the reverse transcription of *cas* genes were carried out with TransScript First-Strand cDNA Synthesis SuperMix (TransGen Biotech Co. Ltd., Beijing, China). CK+ and CK- use the DNA of VD115 and RNase-free water as template, respectively.

**Fig. S8. Genome editing function of the subtype I-C CRISPR-Cas system expressed in *B. thuringiensis*. (A)** Schematic representation of the subtype I-C CRISPR-Cas system-based genome editing. **(B)** Colony PCR detection of the deletion of *cry6Aa.* The 766 and 568 bp bands correspond to the complete and the edited *cry6Aa* gene, respectively.

**Fig. S9. Biofilm formation of BMB1685, BMB1684 and YBT1518 in 96-well.** In brief, 200 μl of a 1:100 dilution of overnight bacterial culture in LB medium was placed in each well. After 48 h of incubation at 28°C, the 96-well was photographed, followed by biofilm formation assays. The turbidity of BMB1685 is apparently lower than that of BMB1684 and YBT1518. Each strain repeats five times.

**Fig. S10**. **Impact of active CRISPR-Cas system on the cell growth of *B. thuringiensis* BMB171 under different environmental stresses. (A)** Growth curve of *B. thuringiensis* strain transformed with subtype I-C CRISPR-Cas system (BMB4072) or same *B. thuringiensis* strain without transformation (BMB171) or transformed with empty vector (BMB1682). The optical absorbance value at 600 nm of these *B. thuringiensis* strains grown in LB medium was analyzed successively from 0 to 54 h. **(B)** Sporulation rate. Sporulation rates were calculated by the CFU of spore-containing aliquots heated at 80°C for 10 min divide by CFU of the aliquots that were kept on ice before growing in LB-agar plates for 24 h at 28°C. **(C)** Biofilm formation ability. The data are expressed as the absorbance at 595 nm of wells treated with different *B. thuringiensis* strains after staining**. (D)** Salt tolerance. The data are expressed as survival rate of the different *B. thuringiensis* strains exposed to salt environments containing 1%, 2%, 4% and 6% NaCl. **(E)** pH tolerance. The data are expressed as survival rate of *B. thuringiensis* strains exposed to different pH environments. In all assays, BMB1682 containing empty pBAC44 vector was used as control**.**

**Fig. S11. Transcription analysis of *gatB* and *rpoA* genes in *B. cereus* group strains.** The RNA was extracted with TranZolΜP Plus RNA Kit (TransGen Biotech Co. Ltd., Beijing, China), and the expression of *gatB and rpoA* genes was detected by qPCR carried out with TransScript First-Strand cDNA Synthesis SuperMix (TransGen Biotech Co. Ltd., Beijing, China). The expression levels of *gatB* gene for each strain at different growth stages were used as reference and set as 1. The relative expression of *rpoA* gene is shown. The differences of recombinant strains against its wild type strains (YBT1518 or BMB171) were evaluated using two-sample *t*-test, lacking of any symbol and ND indicates no statistically significant differences.

**Fig. S12. Number of anti-phage systems in selected *B. cereus* *s. l.* strains with complete and incomplete CRISPR-Cas systems.** The prediction of anti-phage systems was performed through BLASTP using genome-wide amino acid sequences of selected *B. cereus* *s. l.* strains. The number of known DISARM (A), Druantia (B), Gabija (C), Lamassu (D), Septu (E), Thoeris (F) and Zorya-I (G) anti-phage systems per genome were compared among strains with complete and incomplete CRISPR-Cas systems. The detail information of these anti-phage systems is shown in Table S11. The significance of differences among samples was evaluated using two sample Wilcoxon-Mann-Whitney test at *p* < 0.01 (**) and *p* < 0.001 (***). The lack of any symbol indicates no significant difference.

**Fig. S13. The differences of number of virulence genes per genome between *B. thuringiensis* strains with complete, incomplete and no CRISPR-Cas systems.** The prediction of virulence genes was performed through BLASTP using genome-wide amino acid sequences of selected *B. thuringiensis* strains with complete, incomplete and no CRISPR-Cas systems against known virulence factors from reference strain HD-1 [8], with an identity threshold of 30% and dual coverage over 60%. The significance of differences among samples was evaluated using two sample Wilcoxon-Mann-Whitney test at *p* < 0.05 (*), *p* < 0.001 (***) and *p* < 0.0001 (****).

**Supplementary Tables:**

**Table S1. CRISPR-Cas system in *B. cereus* *s. l.* group.**

(This table was submitted directly as an Excel file (designated Table S1) because it contains a large amount of data that cannot be easily shown in a Word file.)

**Table S2. Survey of ecological niches occupied by 423 strains of *B. cereus* *s. l.* group.**

(This table was submitted directly as an Excel file (designated Table S2) because it contains a large amount of data that cannot be easily shown in a Word file.)

**Table S3. Distribution of transposase genes in *B. cereus* *s. l.* group.**

(This table was submitted directly as an Excel file (designated Table S3) because it contains a large amount of data that cannot be easily shown in a Word file).

**Table S4. Replication essential proteins in *B. cereus* *s. l.* group.**

(This table was submitted directly as an Excel file (designated Table S4) because it contains a large amount of data that cannot be easily shown in a Word file).

**Table S5. Prophage proteins in *B. cereus* *s. l.* group.**

(This table was submitted directly as an Excel file (designated Table S5) because it contains a large amount of data that cannot be easily shown in a Word file).

**Table S6. Unique allelic diversity in *B. cereus* *s. l.* group with subtype I-C CRISPR-Cas systems.**

(This table was submitted directly as an Excel file (designated Table S6) because it contains a large amount of data that cannot be easily shown in a Word file).

**Table S7. Bacterial strains and plasmids used in this study.**

| **Strains or plasmids** | **Characteristics** | **Source or reference** |
| --- | --- | --- |
| ***Bacillus thuringiensis*** | | |
| YBT1518 | Wild type strain isolated from China soil sample, that has high activity against nematodes and contains nematicidal *cry5Ba*, *cry6Aa* and *cry55Aa*. | [9] |
| BMB1684 | Derivative of YBT-1518 containing plasmid pBAC44. | This study |
| BMB1685 | Derivative of YBT-1518 containing plasmid pBMB1685. | This study |
| CT-43 | Wild type strain isolated from China soil sample, that has high activity against nematodes and produce nematicidal compound thuringiensis. | [10] |
| BMB171 | No plasmid mutant, acrystalliferous Bt strain | [11] |
| BMB1682 | Derivative of BMB171 containing plasmid pBAC44. | This study |
| BMB4072 | Derivative of BMB171 containing plasmid pBMB1685. | This study |
| ***Bacillus cereus*** | | |
| VD115 | Wild type strain with a complete type I-C CRISPR-Cas system. | From Professor Jacques Mahillon |
| ***Escherichia coli*** | | |
| DH5α | F^-^ φ80*lac*ZΔM15 Δ(*lac*ZYA-*arg*F) U_169_ *end*A1 *rec*A1 *hsd*R17(r_k_^-^, m_k_^+^) *sup*E44λ^-^ *thi*^-1^ *gyr*A_96_ *rel*A1 *pho*A | Thermofisher Scientific Inc., China |
| OP50 | An uracil auxotroph strain whose growth is limited on Nematode Growth Medium (NGM) plates | *Caenorhabditis* Genetics Center |
| DH10B | F^-^ *mcr*A Δ(*mrr*-*hsd*RMS-*mcr*BC) φ80*lac*ZΔM15 Δ*lac*X74 *rec*A1 *end*A1 *ara*D139 Δ(*ara*, *leu*)7697 *gal*U *gal*K λ^-^ *rps*L *nup*G/pMON14272/pMON7124 | Thermofisher Scientific Inc., China |
| pHT304-TTC/DH5α | Derivative of DH5α containing plasmid pHT304-TTC, Amp^r^, Erm^r^. | This study |
| pHT304-TTA/DH5α | Derivative of DH5α containing plasmid pHT304-TTA, Ampr, Ermr. | This study |
| pHT304-TTG/DH5α | Derivative of DH5α containing plasmid pHT304-TTG, Amp^r^, Erm^r^. | This study |
| pHT304-TTT/DH5α | Derivative of DH5α containing plasmid pHT304-TTT, Amp^r^, Erm^r^. | This study |
| pHT304-TGC/DH5α | Derivative of DH5α containing plasmid pHT304-TGC, Amp^r^, Erm^r^. | This study |
| pHT304-TAC/DH5α | Derivative of DH5α containing plasmid pHT304-TAC, Amp^r^, Erm^r^. | This study |
| pHT304-GTC/DH5α | Derivative of DH5α containing plasmid pHT304-GTC, Amp^r^, Erm^r^. | This study |
| pHT304-ATC/DH5α | Derivative of DH5α containing plasmid pHT304-ATC, Amp^r^, Erm^r^. | This study |
| **plasmids** | | |
| pHT304 | An *E. coli* to *B. thuringiensis* shuttle vector, Amp^r^, Erm^r^, 6.6 kb. | [3] |
| pBAC44 | An *E. coli* to *B. thuringiensis* shuttle vector, Cm^r^, Kan^r^, 14 kb. | [1] |
| pHT304-TTC | Derivative of pHT304 containing plasmid PAM site TTC, Amp^r^, Erm^r^. | This study |
| pHT304-TTA | Derivative of pHT304 containing plasmid PAM site TTA, Amp^r^, Erm^r^. | This study |
| pHT304-TTG | Derivative of pHT304 containing plasmid PAM site TTG, Amp^r^, Erm^r^. | This study |
| pHT304-TTT | Derivative of pHT304 containing plasmid PAM site TTT, Amp^r^, Erm^r^. | This study |
| pHT304-TGC | Derivative of pHT304 containing plasmid PAM site TGC, Amp^r^, Erm^r^. | This study |
| pHT304-TAC | Derivative of pHT304 containing plasmid PAM site TAC, Amp^r^, Erm^r^. | This study |
| pHT304-GTC | Derivative of pHT304 containing plasmid PAM site GTC, Amp^r^, Erm^r^. | This study |
| pHT304-ATC | Derivative of pHT304 containing plasmid PAM site ATC, Amp^r^, Erm^r^. | This study |
| pBMB1685 | Derivative of pBAC44 containing Type I-C CRISPR-Cas system from VD115, Cm^r^, Kan^r^ | This study |

**Table S8. The representative targets of spacers on Chromosome genes.**

| **The targets against Chromosome genes** | **Number** |
| --- | --- |
| hypothetical protein | 48 |
| collagen-like protein | 30 |
| MoxR family ATPase | 18 |
| cell division protein FtsK, *Bacillus mycoides* strain Gnyt1 | 15 |
| single-stranded-DNA-specific exonuclease RecJ | 13 |
| NADH dehydrogenase subunit C, *Bacillus thuringiensis* serovar kurstaki str. HD73 | 10 |
| phage infection protein | 10 |
| BclA protein, *Bacillus cereus* strain FORC60 | 9 |
| collagen-like triple helix repeat-containing protein, *Bacillus cereus* strain HBL-AI chromosome | 8 |
| PBP1A family penicillin-binding protein | 8 |
| enterotoxin, *Bacillus anthracis* strain MCCC 1A02161 chromosome | 7 |
| Gly-Xaa-Xaa repeat protein, Bacillus sp. COPE52 | 6 |
| LPXTG cell wall anchor domain-containing protein, *Bacillus cereus* strain ZB201708 chromosome | 6 |
| YhgE/Pip domain-containing protein | 6 |
| cell wall anchor protein, *Bacillus cereus* strain TG1-6 | 5 |
| NAD(P)H-quinone oxidoreductase subunit J, *Bacillus anthracis* CZC5 DNA | 5 |
| penicillin-binding protein | 5 |
| Flagellar hook-length control protein FliK, *Bacillus cereus* strain FORC_047 | 4 |
| **In total** | **288** |

**Table S9. Primer sequences used in this study.**

| **Primers** | **Sequence (5'→3' )*^a^*** | **Functions** |
| --- | --- | --- |
| 16S-F | AGACACGGCCCAGACTCCTAC | To analysis the transcription of 16S rRNA in VD115. |
| 16S-R | GACCCGAAAGCCTTCATCACT |  |
| PAMTTC-S | CGGAATTCGTTTCTTTCACTCCATATGGTA | To verify the PAM site TTC. |
| PAMTTA-S | CGGAATTCTTATTTCACTCCATATGGTA | To verify the PAM site TTA. |
| PAMTTG-S | CGGAATTCTTGTTTCACTCCATATGGTA | To verify the PAM site TTG. |
| PAMTTT-S | CGGAATTCTTTTTTCACTCCATATGGTA | To verify the PAM site TTT. |
| PAMTGC-S | CGGAATTCTGCTTTCACTCCATATGGTA | To verify the PAM site TGC. |
| PAMTAC-S | CGGAATTCTACTTTCACTCCATATGGTA | To verify the PAM site TAC. |
| PAMGTC-S | CGGAATTCGTCTTTCACTCCATATGGTA | To verify the PAM site GTC. |
| PAMATC-S | CGGAATTCATCTTTCACTCCATATGGTA | To verify the PAM site ATC. |
| PAM-A | CCCAAGCTTACAATCACGCCTCAAACA | To verify the PAM site. |
| 6A-up-S | GGGGTACCCGACTTTACCTAGACATTC | To amplify the upstream of *cry6Aa* gene for deletion. |
| 6A-up-A | TCCAAATCATGCTGAGCAGAATGAGCTGGACTACTTTCCC |  |
| 6A-down-S | GGGAAAGTAGTCCAGCTCATTCTGCTCAGCATGATTTGGA | To amplify the downstream of *cry6Aa* gene for deletion. |
| 6A-down-A | GGGGTACCCAAGCTTGATTCCTCCAATAAGTCTC |  |
| del6A-S | GTTGCTGGTGATCCTTCT | To verify the deletion of *cry6Aa* gene. |
| del6A-A | GCCACAACTAACCAAGCA |  |
| crRNA | GCTCTAGA**GTCGCACCTCTTATAGGTGCGTGGATTGAAATTTGAAA ATACTGCTGTTCAGCATATAAAAAATCAAGTCGCACCTCTTATAGG TGCGTGGATTGAAATCAAGAAGGTGACGTGTTAATGGATTGGACG GCCAAGTCGCACCTCTTATAGGTGCGTGGATTGAAAT**GGTACCCC | Synthesized spacer, containing repeat (blue) + spacer1 (red) + repeat (blue) + spacer2 (pink) + repeat sequence, used for forming functional crRNA after being cut. |
| VD115-1S | CGGGATCCGAAGTAATCGTTGCCGTG | To clone F1 fragment of VD115 CRISPR-Cas system |
| VD115-1A | GCTCCACATATCCTTGACA |  |
| VD115-2S | GCAGCCGAACATTTCAAC | To clone F2 fragment of VD115 CRISPR-Cas system |
| VD115-2A | AACCGCTACACCTACTTCC |  |
| VD115-3S | CGACCAGGCAGAGTATCT | To clone F3 fragment of VD115 CRISPR-Cas system |
| VD115-3A | CGGGATCCTACACATTCACCGAGGATG |  |
| *^a^* Restriction sites are underlined. Red bases in primers, such as PAMTTC-S are PAM sites. The blue sequences in crRNA are repeats, the red sequence is a spacer from target *cry6Aa* gene, and the pink sequence is a spacer from original CRISPR-Cas system of VD115. **Table S10. Bioinformatics of six cloned protospacers and 5'-TTC PAM site.** **spacer number** \| **5 ' flank** \| **protospacer sequence** \| **3' flank**; VD115_GCA_000290835.1_JH792172.1_tmp_8_Crispr_1_spacer8 \| GT**TTC** \| ATAGATGGTGACGGCACTGGATTACAACACATATT \| TGTTT; VD115_GCA_000290835.1_JH792172.1_tmp_8_Crispr_1_spacer10 \| GT**TTC** \| ATGGTTTCGAATATTATCAAGAATAGCTTTACT \| TGTTT; VD115_GCA_000290835.1_JH792172.1_tmp_8_Crispr_1_spacer13 \| GT**TTC** \| AGAACAATTATTAGTTAGAATCGGAGACCCCGGTG \| TGTTT; VD115_GCA_000290835.1_JH792172.1_tmp_8_Crispr_1_spacer14 \| GT**TTC** \| ACAATTTTATCAAAATAAGAAAAAACTATTTGA \| TGTTT The blue bases are PAM sites. **Table S11. Prediction of anti-phage systems in *B. cereus* *s. l.* group.** (This table was submitted directly as an Excel file because it contains a large amount of data that cannot be easily shown in a Word file). | | |

**Table S12. Sequence analysis of PAM sites of subtype I-C CRISPR-Cas system in *B. cereus* VD115.**

| **Spacer number** | **Order** | **Target genome** | **5' flank** | **Protospacer sequence** | **3' flank** |
| --- | --- | --- | --- | --- | --- |
| CR1_ spacer1 | S1851 | MC28 plasmid pMC95 | TT**TTC** | TTTCACTCCATATGGTAAACCACGACTCATTTTA | TGTTT |
|  |  | CT43 plasmid pCT83 | GT**TTC** | TTTCACTCCATATGGTAAACCACGACTCATTTTA | TGTTT |
|  |  | YBT-1518 plasmid pBMB0233 | GT**TTC** | TTTCACTCCATATGGTAAACCACGACTCATTTTA | TGTTT |
|  |  | IS5056 plasmid pIS56-85 | GT**TTC** | TTTCACTCCATATGGTAAACCACGACTCATTTTA | TGTTT |
|  |  | AH187 plasmid pAH187_45 | GT**TTC** | TTTCACTCCATATGGTAAACCACGACTCATTTTA | TGTTT |
| CR1_ spacer7 | S1018 | *Bacillus* phage250 | GG**TTC** | AAACATAGGGTTAACATTGCTCATGATGCTCTCC | CCTTT |
| CR1_ spacer8 | S442 | *Bacillus* phage BMBtp9 | TA**TTT** | ATAGATGGTGACGGCACTGGATTACAGCACATATT | CGGTA |
|  |  | *Bacillus* phage250 | TA**TTT** | ATAGATGGTGACGGCACTGGATTGCAACACATATT | TGGTG |
| CR1_spacer12 | S1874 | H3081.97 plasmid pH308197_29 | AT**TTC** | TTTGGATTAGCAAATGATGATTACACCGTTGAA | AAAAT |
| CR2_ spacer4 | S285 | MC28 plasmid pMC183 | CA**TTC** | ACGTATATATGTTTTTCCTTTGAGAAACCCTTAGT | ATTAAT |
|  |  | G9842 plasmid pG9842_209 | CA**TTC** | ACGTATATATGTTTTTCCTTTGAGAAACCATTAGT | TTTAA |

The bold font indicates the predicted PAM sites for subtype I-C CRISPR-Cas system in *B. cereus* VD115.

**Supplementary Datasets:**

**Dataset 1. Graphic representation of CRISPR spacers in *B. cereus* *s. l.* group.**

(This dataset was submitted directly as an Excel file (designated Dataset 1) because it contains a large amount of data that cannot be easily shown in a Word file).

**Dataset 2. Targets of representative CRISPR-Cas spacers in *B. cereus* *s. l.* group.**

(This dataset was submitted directly as an Excel file (designated Dataset 2) because it contains a large amount of data that cannot be easily shown in a Word file).

**Supporting References:**

1.	Liu XY, Ruan LF, Hu ZF, Peng DH, Cao SY, Yu ZN, et al. Genome-wide screening reveals the genetic determinants of an antibiotic insecticide in *Bacillus thuringiensis*. *J Biol Chem.* 2010;285:39191-200.

2.	Peng D, Luo Y, Guo S, Zeng H, Ju S, Yu Z, et al. Elaboration of an electroporation protocol for large plasmids and wild-type strains of *Bacillus thuringiensis*. *J Appl Microbiol*. 2009;106:1849-58.

3.	Arantes O, Lereclus D. Construction of cloning vectors for *Bacillus thuringiensis*. *Gene*. 1991;108:115-9.

4.	Nam KH, Haitjema C, Liu X, Ding F, Wang H, DeLisa MP, et al. Cas5d protein processes pre-crRNA and assembles into a cascade-like interference complex in subtype I-C/Dvulg CRISPR-Cas system. *Structure*. 2012;20:1574-84.

5.	Zheng J, Peng D, Ruan L, Sun M. Evolution and dynamics of megaplasmids with genome sizes larger than 100 kb in the *Bacillus cereus* group. *BMC Evol Biol*. 2013;13:262.

6.	Price MN, Dehal PS, Arkin AP. FastTree: computing large minimum evolution trees with profiles instead of a distance matrix. *Mol Biol Evol*. 2009;26:1641-50.

7.	Grissa I, Vergnaud G, Pourcel C. CRISPRFinder: a web tool to identify clustered regularly interspaced short palindromic repeats. *Nucleic Acids Res*. 2007;35:W52-7.

8.	Zhu L, Peng D, Wang Y, Ye W, Zheng J, Zhao C, et al. Genomic and transcriptomic insights into the efficient entomopathogenicity of *Bacillus thuringiensis*. *Sci Rep*. 2015;5:14129.

9.	Wang P, Zhang C, Guo M, Guo S, Zhu Y, Zheng J, et al. Complete genome sequence of *Bacillus thuringiensis* YBT-1518, a typical strain with high toxicity to nematodes. *J Biotechnol*. 2014;171:1-2.

10.	He J, Wang J, Yin W, Shao X, Zheng H, Li M, et al. Complete genome sequence of *Bacillus thuringiensis* subsp. *chinensis* strain CT-43. *J Bacteriol*. 2011;193:3407-8.

11.	He J, Shao X, Zheng H, Li M, Wang J, Zhang Q, et al. Complete genome sequence of *Bacillus thuringiensis* mutant strain BMB171. *J Bacteriol*. 2010;192:4074-5.
